# Single trait versus principal component based association analysis for flowering related traits in pigeonpea

**DOI:** 10.1038/s41598-022-14568-1

**Published:** 2022-06-21

**Authors:** Kuldeep Kumar, Priyanka Anjoy, Sarika Sahu, Kumar Durgesh, Antara Das, Kishor U. Tribhuvan, Amitha Mithra Sevanthi, Rekha Joshi, Pradeep Kumar Jain, Nagendra Kumar Singh, Atmakuri Ramakrishna Rao, Kishor Gaikwad

**Affiliations:** 1 ICAR-National Institute for Plant Biotechnology, New Delhi, India; 2 ICAR-Indian Institute of Pulses Research, Kanpur, Uttar Pradesh India; 3 ICAR-Indian Agricultural Statistics Research Institute, New Delhi, India; 4 Division of Genetics, ICAR-Indian Agricultural Research Institute, New Delhi, India; 5 ICAR-Indian Institute of Agricultural Biotechnology, Ranchi, Jharkhand India

**Keywords:** Biotechnology, Plant sciences

## Abstract

Pigeonpea, a tropical photosensitive crop, harbors significant diversity for days to flowering, but little is known about the genes that govern these differences. Our goal in the current study was to use genome wide association strategy to discover the loci that regulate days to flowering in pigeonpea. A single trait as well as a principal component based association study was conducted on a diverse collection of 142 pigeonpea lines for days to first and fifty percent of flowering over 3 years, besides plant height and number of seeds per pod. The analysis used seven association mapping models (GLM, MLM, MLMM, CMLM, EMLM, FarmCPU and SUPER) and further comparison revealed that FarmCPU is more robust in controlling both false positives and negatives as it incorporates multiple markers as covariates to eliminate confounding between testing marker and kinship. Cumulatively, a set of 22 SNPs were found to be associated with either days to first flowering (DOF), days to fifty percent flowering (DFF) or both, of which 15 were unique to trait based, 4 to PC based GWAS while 3 were shared by both. Because PC1 represents DOF, DFF and plant height (PH), four SNPs found associated to PC1 can be inferred as pleiotropic. A window of ± 2 kb of associated SNPs was aligned with available transcriptome data generated for transition from vegetative to reproductive phase in pigeonpea. Annotation analysis of these regions revealed presence of genes which might be involved in floral induction like Cytochrome p450 like Tata box binding protein, Auxin response factors, Pin like genes, F box protein, U box domain protein, chromatin remodelling complex protein, RNA methyltransferase. In summary, it appears that auxin responsive genes could be involved in regulating DOF and DFF as majority of the associated loci contained genes which are component of auxin signaling pathways in their vicinity. Overall, our findings indicates that the use of principal component analysis in GWAS is statistically more robust in terms of identifying genes and FarmCPU is a better choice compared to the other aforementioned models in dealing with both false positive and negative associations and thus can be used for traits with complex inheritance.

## Introduction

The United Nations 2nd Sustainable Development Goal (SDG-2) aims to eradicate hunger and malnutrition globally by 2030. The goal has become even more challenging in the current context of the Covid-19 pandemic, which has devastating effect on agricultural sector that by 2030, the number of hungry peoples may exceed 840 million, with the majority (above 381 million) from the Asian (https://www.un.org/sustainabledevelopment/hunger/) region. However in order to reach SDG-2 standards and commitments, it is necessary to prioritize nutrition in addition to food security. Pulses are important in combating malnutrition, as in addition to providing a sustainable production system, they are the crucial component of human diet (http://www.fao.org/resources/infographics/).

Pigeonpea (*Cajanus cajan* (L.) Millsp.), is a highly nutritious grain legume. Although it is a perennial plant, but primarily cultivated as an annual crop with sowing to flowering duration ranging between 60 to 180 days. While long duration varieties have higher yield potential, lately, a significant shift towards shorter duration varieties has occurred so as to accommodate them in diverse cropping systems. Hence, the development of short duration varieties with comparable yield potential is compelling need of the hour. Cultivation of short duration varieties also enables farmers to escape adverse growth conditions, such as drought, severe winter, and disease incidences. The main trait that can be targeted for developing varieties with a specific duration is the number of days to flowering. Thus, biotechnological interventions could be deployed to speed up the development of short-duration varieties^1–4^.

Floral development is a definitive event in the evolution of flowering plants; interestingly no non flowering mutants have been identified to date, and researchers have only been able to alter the days to flowering in plants by modifying a few gene combinations. Floral transition is controlled at both pre and post translation levels^5,6^. Autonomous pathway, vernalization, light dependent floral induction, hormonal control and starch dependent controls are the major floral induction pathways. *FLC*,* SOC1*,* FVE*,* FLD*,* CO*,* FT* are just few of the critical genes involved in this process^7^, while auxin and gibberellic acid serve as the primary hormonal regulators of floral transition^8^. The floral transition phenomenon has been extensively investigated in *Arabidopsis* and a few legumes^9,10^. So far, 306 genes regulating floral development have been characterised in *Arabidopsis*^10^.

To develop shorter duration varieties of pigeonpea, it is essential to understand the mechanisms underlying flowering time variations and its adaptation to different ecologies. Though efforts have been made to characterise the *MADS* box genes, *PEBP* gene family, *CCT* gene family, lncRNAs influencing floral induction and to map the loci governing earliness and domestication related traits, but the genes and markers associated with days to flowering are not yet known in pigeonpea^11–16^. Due to the fact that pigeonpea has four maturity groups^17^, Genome Wide Association Studies (GWAS) can better capture the genetic basis of flowering than bi-parental mapping populations since it uses the natural population that represents all allelic combinations arising out of historical recombinations. The GWAS approach has been shown to be effective in identifying novel genes and QTLs for multiple traits in diverse germplasm of rice and wheat^18,19^. The availability of a 62 K SNP chip and hyper variable markers covering the complete pigeonpea genome, together with low cost sequencing costs, enables efficient GWAS analysis in pigeonpea^20–24^.

Previous reports on GWAS in several legume crops have focused on domestication related loci, resistance against fusarium wilt, and days to flowering in chickpea; days to flowering and maturity in soybean, but a computationally robust analysis is still needed to decipher association and develop markers with high confidence for flowering related traits in pigeonpea^12,25–27^. The current work used an association panel of 142 accessions in order to identify candidate genes and markers for flowering-related traits in pigeonpea.

Principal Component Analysis (PCA) is a powerful dimension reduction and an unsupervised linear transformation technique which aims to extract critical information from phenotypically complex traits while reducing the redundancy in variables and preserving the information parallelly. It reduces a large set of initially correlated variables to a much smaller set of uncorrelated or orthogonal variables termed as PCs. GWAS using PC scores as dependent variables are more reliable and robust than single trait based, and it can reveal possible pleiotropy with increased power^18,28^. As a result, we conducted a PCA based GWAS to discover genetic factors regulating crop architecture with emphasis on flowering. The effectiveness of the approach in identifying significant genes associated with pigeonpea flowering and related traits was further validated through annotation of the flanking regions using transcriptome data of ICPL 20338 accession (PRJNA752250). Thus, the present study was undertaken with the following objectives: (i) Single trait based GWAS using seven association mapping models (GLM, MLM, MLMM, CMLM, EMLM, FarmCPU and SUPER) to identify novel genes to flowering and related traits (ii) PC based GWAS to improve the accuracy and robustness of single trait GWAS and investigate pleiotropy (iii) Genomic prediction and (iv) Annotation of significantly associated SNPs.

## Material and methods

### Plant material, phenotyping and ANOVA

A collection of 142 accessions representing a global pigeonpea germplasm collection, which includes landraces and breeding lines (mostly of Indian origin) (Table S1), were procured and maintained in the Division of Genetics, Indian Agricultural Research Institute (ICAR), New Delhi, India. These lines were grown using the recommended package of practises for 3 years (2017–18 to 2019–20) in replicates. Data for four quantitative traits, i.e. days to first flowering (DOF), days to fifty percent flowering (DFF), plant height (PH) and average number of seeds/pod (SPP) were taken from each individual line in 2017–18 and 2018–19 and average values from the replicates were used for analysis. In 2019–20, data was collected solely for DOF and DFF traits. Days required to develop one completely opened flower in any plant of a row was noted as DOF, while days required by minimum 50% of plants in a row to have one open flower was noted as DFF. The PH of the plant was noted on maturity considering the last twig as end point, whereas SPP was measured by taking the average of randomly selected 50 pod and rounding off till one decimal point. The basic statistics of the phenotypic data were calculated through the STAR (Statistical Tool for Agricultural Research) tool available at http://bbi.irri.org/products. Analysis of variance and broad sense heritability of the data was calculated using Indostat (version 8.1). The whole study complies with relevant institutional, national, international guidelines and legislation.

## Reference based assembly of all lines

The raw sequence data of all 142 lines used in the present study is available at NCBI (https://www.ncbi.nlm.nih.gov/). These datasets were downloaded and processed to remove adapters and poor quality reads through Trimmomatic version 0.36^29^ using default parameters. High quality reads were mapped to the pigeonpea reference genome of cultivar ICPL 87119 available at NCBI using the BWA tool version 0.7.17^21,30^. Samtools version 1.10 and Freebayes version 1.3.1 were used further for variant calling with minimum 10 × depth as basic criteria^31,32^. InDels were removed and SNPs covering minimum of 80% reads were filtered. All 142 .vcf files generated were merged to a single .vcf file through samtools, which was used to develop hapmap through Tassel 5.0^33^. SNPs that showed a Missing Allele Frequency of more than 0.05 were not considered for Hapmap preperation, which ultimately included 168,540 SNPs.

### Population structure analysis

Population structure is known to affect association studies, but we need to look after its impact. The power to detect population structure is highly dependent on the number of loci utilised^34,35^. Furthermore, increased heterogeneity may lead to false stratification^36,37^. Thus, the combined variant file was filtered for 1 SNPs within a sliding window of 200 kb with maximum depth and minimum number of missing samples and eventually 1229 SNPs out of 168,540 were selected for structure analysis. FastSTRUCTURE v1.0^38^ was used to investigate the population structure of all 142 accessions. The number of groups/sub-populations (k) was set from 1 to 10 with the burn-in period, and the number of Markov Chain Monte Carlo (MCMC) replications after burn-in were both set to 100,000 under the “admixture mode”. Five independent runs were performed for each k number. Finally, the structure was developed using the STRUCTURE harvester vA.2^39^. The delta K method developed by Evanno et al.^40^ was used to determine the optimal value of k.

### Genetic diversity estimation

The diversity estimation was done using Tassel 5.0^33^ based on the nucleotide diversity (π), Watterson estimator (θ), and Tajima’s D index^41–43^.

### Principal component analysis

PCA was conducted using R software version 4.0.0. Reduced DOF and DFF, but increased SPP, are desirable for the generation of short duration varieties with higher yield potential. Given the intricate inheritance and genetic correlation of quantitative traits, some trade-off is inevitable. As various quantitative phenotypic variables were measured in different units reflecting different types of interpretations, for statistical validity, the original variables were standardized (with mean 0 and variance 1) before attempting PCA. The detailed description about the PCA statistics and their loadings are provided in Table S2.

### Genome wide association study

GWAS was conducted using the GAPIT package version 3.0 in R software, which employs Bonferroni correction to define statistically significant MTAs. For this study seven association models were implemented namely General Linear Model (GLM), Mixed Linear Model (MLM), Multiple Loci MLM (MLMM), Compressed MLM (CMLM), Enriched CMLM (ECMLM), Fixed and Random Model Circulating Probability Unification (FarmCPU) and Settlement of MLM Under Progressively Exclusive Relationship (SUPER) algorithm^44–50^. These models are distinct in their basic structures and components included as fixed and random effects. Except GLM all the models include mixed effect structures, that is both fixed and random effect components. PC scores were used as covariates or fixed effects in PC based GWAS. CMLM, ECMLM and SUPER generally exhibit higher statistical power as compared to the MLM^51^. Amongst all models, MLMM and FarmCPU algorithms are for multiple loci analysis. FarmCPU model is designed to control both false positives and false negatives as compared to other models^49,51^. Table 1 describes all the association mapping models, also explaining their differences.

**Table 1:** Description of the association mapping models.

Model	Description
General linear model (GLM)	GLM induces the simplest structure for single-locus analysis with population structure (Q) as fixed effect, whereas no random effect component is involved in the model; principal components are used as covariates in such a model to reduce the false positives
Mixed linear model (MLM)	MLM includes the kinship matrix (K) as an additional random effect component; hence it is also called the Q + K model
Multiple loci MLM (MLMM)	MLMM is designed for multiple locus analysis, is an improvement over MLM which incorporates multiple markers simultaneously as covariates in order to partially remove the confounding between testing markers and kinship. Gapit uses forward and backward stepwise linear mixed-model regression to include the markers as covariates
Compressed MLM (CMLM)	In CMLM the similar individuals are assigned into groups through cluster analysis and then groups are used as elements of reduced kinship matrix for random effect structure. The model has improved statistical power compared to regular MLM methods due to grouping or clustering
Enriched CMLM (ECMLM)	ECMLM calculates kinship using different algorithms and then chooses the best combination between kinship algorithms and grouping algorithms
Fixed and random model circulating probability unification (FarmCPU)	This is an iterative approach which iteratively fits both fixed and random effect model to eliminate the models overfitting problem while using stepwise regression in MLMM. To control the false positives, kinship derived from associated markers is used
Settlement of MLM under progressively exclusive relationship (SUPER)	The SUPER model uses the associated genetic markers (pseudo Quantitative Trait Nucleotides) to derive the kinship matrix, instead of all the markers. Whenever a pseudo QTN is correlated with the testing marker, it is excluded from those used to derive kinship. The method has higher statistical power than regular MLM

### Genomic prediction

Genomic prediction (GP) modelling was done through the rrBLUP (v4.6) package in R software^52^ for ridge-regression based genome-wide regression. The Ridge Regression Best Linear Unbiased Prediction (RRBLUP) model for genome-wide regression assumes the following form,

$${\mathbf{y = Xb + Zu}} + {{\varvec{\upvarepsilon}}}$$

Where **y** is the vector of phenotypic values; **X** and **Z** are the design matrices for fixed and random effects respectively; **b** and **u** are the coefficient vectors of fixed and random effects respectively; **u** is assumed to follow normal distribution $N(0,\,{\mathbf{I}}\sigma_{u}^{2} )$ and error term ${{\varvec{\upvarepsilon}}} \sim N(0,\,{\mathbf{I}}\sigma_{\varepsilon }^{2} )$ with **I** being the identity matrix. The random effect coefficient **u** is used to represent the marker effects associated with **Z** being the matrix of genotypes. The variance components $\sigma_{u}^{2}$ and $\sigma_{\varepsilon }^{2}$ are estimated through the Maximum likelihood (ML) or Restricted ML (REML) method.

### Annotation of the associated loci using transcriptome data

For annotation analysis, a window of ± 2 kb was used for each associated SNP for annotation analysis. The selected windows were looked at for similarity searches within the assembled *in-house* transcriptome data of early flowering genotypes (ICPL 20338) (PRJNA752250) present in two biological replicates, through the BLASTn program. Initially the raw reads were filtered through trimmomatic version 0.36^29^ to remove the poor quality reads with default parameters. Cleaned reads were de novo assembled using Trinity (version 2.1.1)^53^ with default parameters. Differentially expressed genes (DEGs) were identified using the edgeR package in the bioconductor environment through R script. These DEGs were filtered on the basis of p value (0.001), FDR < 0.05 and on the basis of fold change of the fragments per kilo-base of transcript per Million fragments mapped (FPKM) value (± 2). CD-hit web server (http://weizhong-lab.ucsd.edu/cd-hit/) was used to remove duplicates. The FPKM values of genes corresponding to vegetative leaves (VL), reproductive leaves (RL), shoot apical meristem (SAM) and reproductive buds (Bud) were retrieved to construct heatmap using Morpheus web server (https://software.broadinstitute.org/morpheus/).

### Ethical approval

This article does not contain any studies with animals performed by any of the authors.

## Results and discussion

### Descriptive statistics of phenotypes and PCs

**Figure 1:**
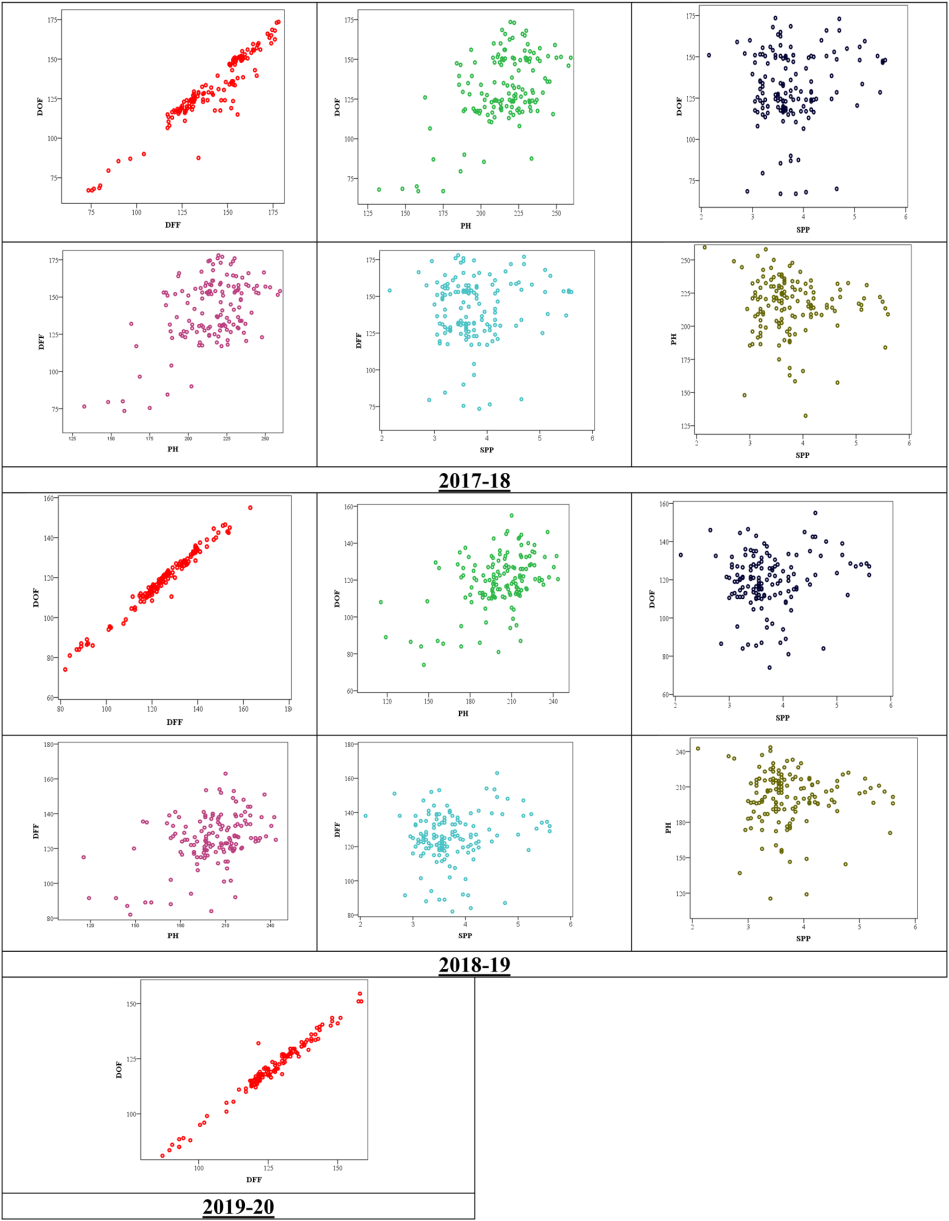
Scatter diagrams showing collinearity among the selected phenotypic traits for different years. Upward linear pattern indicates greater extent of positive correlation. Days to first flowering (DOF), days to fifty percent flowering (DFF), plant height (PH) and average number of seeds/pod (SPP).

The phenotypic data of quantitative traits, namely DOF and DFF, was taken over 3 years in all 142 lines, whereas data for PH and SPP was taken only for 2 years, i.e. 2017–18 and 2018–19. The cumulative descriptive statistics from the first 2 years of phenotypic data are provided (Tables S3 and S4). The value of DOF was varying from 67 to 174 with an average of 125; the value of DFF was varying from 73 to 178 with a mean of 133; PH in the selected lines were ranging from 118 to 261 with an average of 208; SPP was ranging between 2 to 5 with a mean value of 3. The standard deviations of DOF, DFF, PH and SPP were 0.83, 0.85, 1.01 and 0.02 respectively, while the percentage coefficient of variation (CV) value of DOF, DFF, PH and SPP were 15.4, 15.8, 11.6 and 16.6. Evidently, SPP showed maximum variation and PH manifested minimum variability in terms of percentage CV. For 2017–18 data, the Pearson’s linear correlation coefficient between DOF and DFF was 0.95; correlation between DOF and PH was 0.50; DFF and PH had correlation 0.50; whereas, PH and SPP showed negative correlation (Table S4). The same pattern followed in other years too, as depicted in the bi-variate scatter diagram (Fig. 1). Presence of high variability in PH, SPP, DOF and DFF across the year and genotype was observed for all the traits (Table 2). Broad sense heritabiilty (h^2^) of PH, SPP, DOF and DFF were 0.5449, 0.5897, 0.6593 and 0.7094 respectively (Table 2), which is the suggestive that major proportion of the variation is due to difference in genotypes.

**Table 2:** Pooled Analysis of Variance (Pooled-ANOVA) for four traits evaluated in different environments.

Source	PH		SPP		DOF		DFF	
	d.f.	MSS	d.f.	MSS	d.f.	MSS	d.f.	MSS
Year	1	25,764.88***	1	0.07	2	12,345.08***	2	21,545.25***
Genotype	141	1157.32***	141	0.96***	141	907.85***	141	895.31***
Genotype × Year	282	359.84	282	0.29	282	91.81	282	97.91
Broad Sense heritability (*h*^2^)	0.5449		0.5897		0.6593		0.7094	

The correlation among the original variables is suggestive of using PCA and thereby using PC scores for GWAS analysis. For 2017–18, PC1 explained 58% of the variation in the original data, while PC1 and PC2 together explained 85% of the variation; further adding PC3 explained 98% of the variation. Similarly, in 2018–19, PC1 and PC2 determined 57% and 26% variations respectively. We have taken the first two PCs each year to perform GWAS as they preserved the majority of the variations (> 80%) in the original data (Table S2). The first two traits, namely DOF and DFF, exhibited maximum positive loadings on PC1, followed by PH. Loading on PC2 was higher for SPP trait, whereas plant height showed negative loading on PC2; this means PC2 is mainly representative of SPP. The PCs followed normal distribution, exhibiting non-significant results in Shapiro–Wilk test for Normality (Null hypothesis: Data follows Normal distribution), while amongst the single traits, SPP failed to show normality (SPP had count data). Therefore, PC based GWAS is supposed to improve the statistical power of GWAS analysis.

### Genetic diversity and related analysis

The nucleotide diversity (π) is a reflection of genetic diversity which can be used to monitor diversity and genetic variation in crops and related species^54^ or to determine evolutionary relationship. In our analysis, the value of π was 0.03573. The Watterson estimator (θ), which is an estimation of the population mutation rate was 0.19898. Usually both π and θ ranges in between 0–1, where the inclination toward 0 indicates presence of less diversity. Pigeonpea is an often cross pollinated crop but harbours less diversity, especially in the landraces and cultivated varieties due to progressive bottlenecks during domestication and breeding^12^. Our study was based on only 142 landraces and cultivated varieties mainly of Indian origin, and hence the lesser diversity could be explained. Similar results were reported by other groups also^55,56^. The Tajima’s D in our population was − 2.84379. Tajima's D is computed as the difference between two measures of genetic diversity: the mean number of pairwise differences and the number of segregating sites, each scaled so that they are expected to be the same in a neutrally evolving population of constant size. When Tajima’s D value is less than 0, it means abundant rare alleles are present, suggesting a possible selective sweep and population expansion.

### Population structure and kinship analysis

**Figure 2:**
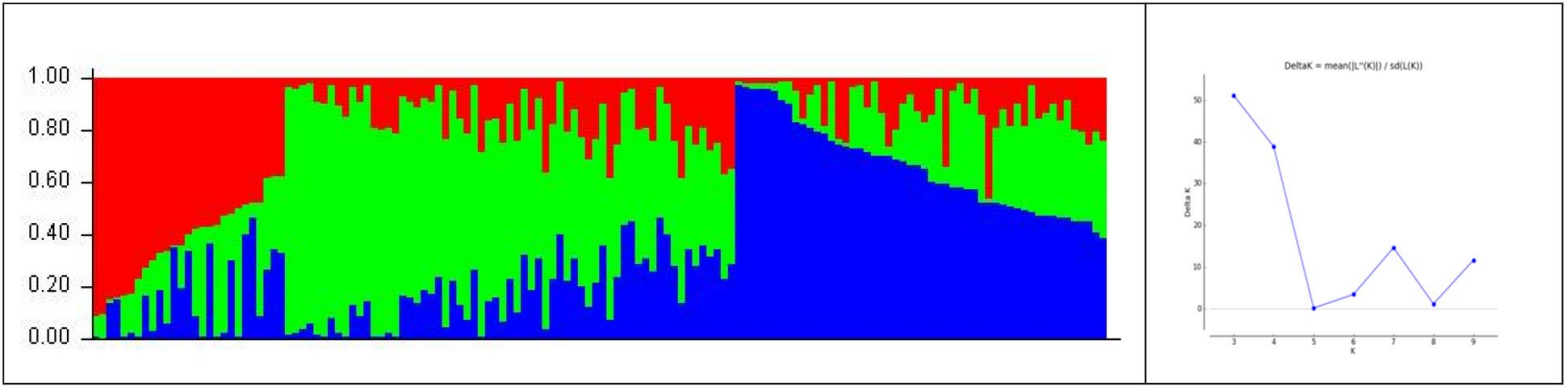
Population structure analysis revealed three major clusters in the pigeonpea mini core collection.

Population structure, kinship analysis, as well as diversity estimates suggested the presence of less diversity among the studied genotypes. The collection was stratified into 3 clusters (k = 3) with a substantial level of admixture, probably the result of its pollination behaviour (often cross pollinated), which is in accordance with previous reports^57^. Cluster 1 comprised 28 accessions, while clusters 2 and 3 comprised 63 and 51 lines respectively, in the population structure analysis (Fig. 2). Similar results were obtained with the kinship matrix where the same clustering pattern was observed (Figure S1).

### GWAS results

**Figure 3:**
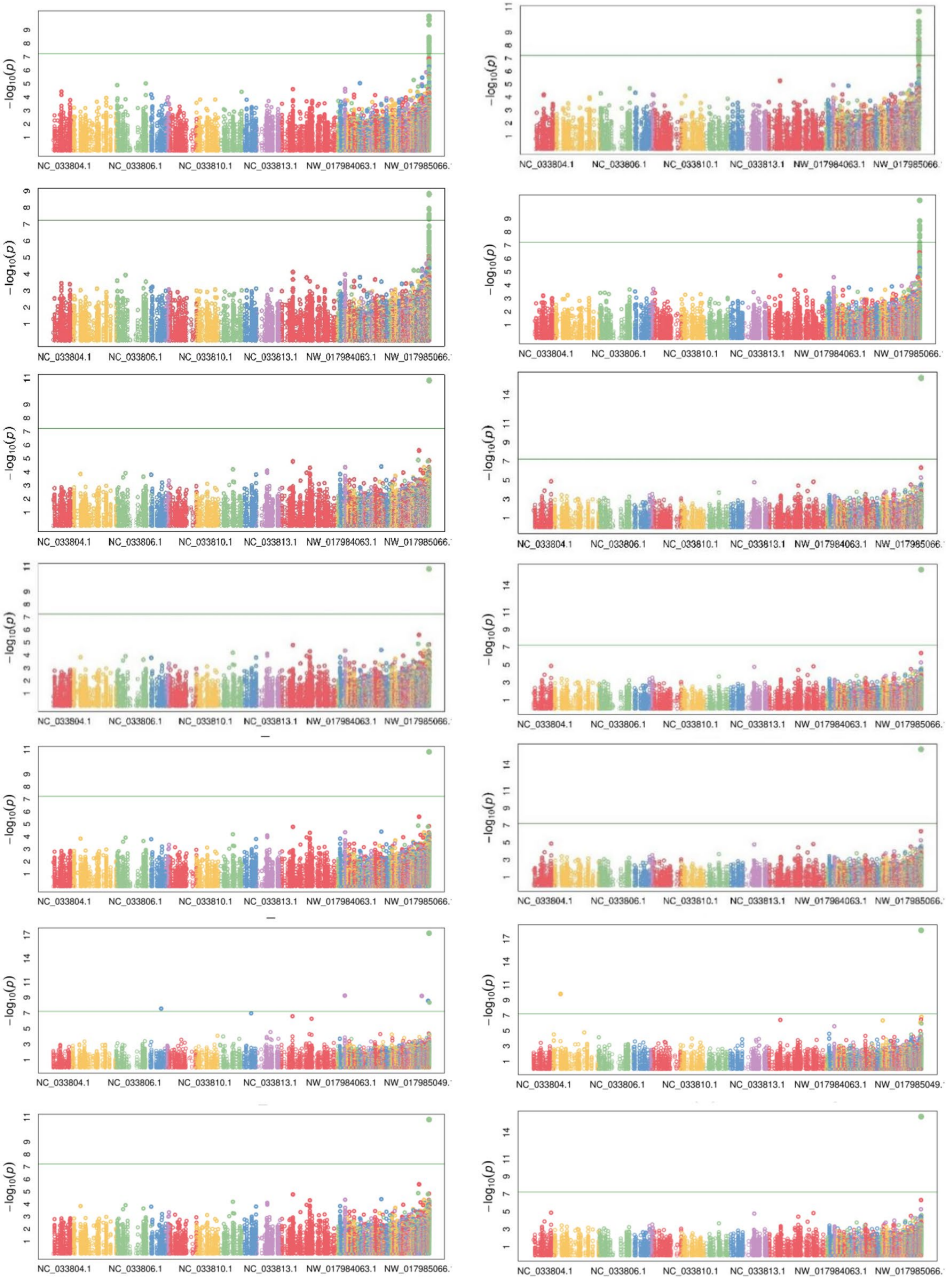
Manhattan plots for DOF (Left side) and DFF (Right side) for the year 2017–18. Top to bottom order is GLM, MLM, MLMM, CMLM, ECMLM, FarmCPU and SUPER.

In all the aforementioned models, GWAS for quantitative traits like DOF and DFF revealed the association of 19 and 22 non-redundant SNPs distributed on chromosomes 2 and 6 respectively, in the 2017–18 data. However, no significant association was found for PH and SPP. A descriptive summary of GWAS on DOF and DFF in 2017–18 is provided in Tables S5-S6 and Fig. 3. GWAS on 2018–19 data revealed the association of 11 and 9 non-redundant SNPs for DOF and DFF, respectively (Tables S7–S8 and Figure S11). However, no significant association was found for PH and SPP in 2018–19, hence they were excluded from phenotyping in 2019–20. In 2019–20, 13 and 10 non redundant SNPs were found to be associated with DOF and DFF, by all seven models (Tables S9–S10 and Figure S12). The first two PCs constructed from 2017–18 and 2018–19 data were then analysed for association. PC based GWAS of PC1 in 2017–18 data revealed an association of 18 non-redundant SNPs, while the same in 2018–19 data showed an association of 12 non-redundant SNPs (Tables S11–S12 & Figure S13 and S14). GWAS on PC2 in 2017–18 gave a total of 6 non-redundant SNPs, though no association was found in PC2 2018–19 data (Table S13). As PC2 reflects the loading of SPP, these loci must be regulating SPP traits.

Cumulatively, trait based and PC1 based GWAS on DOF and DFF revealed the association of 22 SNPs with DOF/DFF traits and were selected for further annotation analysis (Table 3). Interestingly, this set of SNPs also included the SNP 812678807:41, which was consistently found to be associated with both the traits as well as the PCs, across all three-year data by each of the seven models employed (Fig. 3 & S11–14). As the distance between the SNPs approached 1.5 Mb, the average r^2^ was 0.1^58^. This LD level usually indicates that there is nearly no linkage between the markers after this; thus, we defined SNPs within a 1.5 Mb window as a single locus. So all of the associated SNPs scattered on scaffold NW_017988637.1 were treated as a single locus for further analysis as it’s of only 5 kb in size. Out of 24 SNPs 812678863:261: +, 812678807:41: + and 812679326:250: + were on the same scaffold and hence treated as single loci and the whole scaffold was considered for annotation purposes.

**Table 3:** List of selected SNPs further used for annotation analysis.

S. no.	SNP id	Chromosome	Physical location	Year (trait)
1	812678863:261: +	NW_017988637.1	1117	2017–18 (DOF)
2	392468479:318: +	NW_017984071.1	155,917	2017–18 (DOF)
3	725832748:272: +	NW_017985276.1	22,384	2017–18 (DOF)
4	791831919:74: +	NW_017986933.1	11,488	2017–18 (DOF) and 2019–20 (DOF)
5	142343707:25: +	NC_033807.1	9,366,686	2017–18 (DOF)
6	760222832:55: +	NW_017985856.1	27,685	2018–19 (DOF), 2018–19 (DFF) and 2018–19 (PC1)
7	812678807:41: +	NW_017988637.1	869	2018–19 (DOF) and 2019–20 (DOF), 2018–19 (DFF) and 2019–20 (DFF)
8	376936577:87: −	NW_017984062.1	168,305	2018–19 (DOF)
9	652249420:11: +	NW_017984675.1	23,436	2018–19 (DOF)
10	709017214:7: −	NW_017985090.1	533	2018–19 (DOF)
11	633271872:58: +	NW_017984581.1	74,012	2018–19 (DOF)
12	392479221:11: +	NW_017984071.1	161,167	2019–20 (DOF), 2019–20 (DFF), and 2018–19 (PC1)
13	164755426:8: +	NC_033809.1	6,932,346	2019–20 (DOF) and 2019–20 (DFF)
14	781124881:96: −	NW_017986454.1	6679	2019–20 (DOF)
15	812679326:250: −	NW_017988637.1	863	2017–18 (DFF), 2017–18 (PC1)
16	35373484:284: +	NC_033805.1	6,362,335	2017–18 (DFF)
17	785047004:88: +	NW_017986607.1	3977	2018–19 (DFF)
18	330539130:289: +	NC_033814.1	21,328,862	2019–20 (DFF)
19	21256769:324: +	NC_033804.1	14,401,967	2017–18 (PC1)
20	740074801:308: −	NW_017985477.1	249	2017–18 (PC1)
21	324910270:94: −	NC_033814.1	17,612,083	2018–19 (PC1)
22	593701379:271: +	NW_017984430.1	87,462	2018–19 (PC1)

Three SNPs (760222832:55: +, 392479221:11: + and 812679326:250: −) were found to be associated with both PC1 and either DOF/DFF or both, while four SNPs (21256769:324: +, 740074801:308: −, 324910270:94: − and 593701379:271: +) were found to be associated exclusively with PC1 scores, reflecting the ability of PC based GWAS in identifying novel associations. As PC1 is the representative of DOF, DFF and PH, these unique SNPs might be regulating plant height in addition to DOF and DFF. As PH is arrested once flowering starts in the case of determinate lines, the role of these SNPs in regulating plant height can’t be ignored. Further, among these novel associations identified by PC, 593701379:271 was annotated as F box protein (Table 4), which is a vital component of auxin signalling playing an important role in vegetative to reproductive phase transition as well as in determining plant height.

**Table 4:** Annotation of the SNPs showing marker trait association reveals role of auxin pathway genes in flower induction.

S. no.	SNP id	Putative candidate regulators in 2 kb window of associated SNPs
1.	812678863:261: +	Transcript (TRINITY_DN34349_c0_g1_i9) annotated as cytochrome P450-like TATA box binding protein (cytochrome P450-like TBP)
2.	812679326:250: +	
3.	812678807:41: +	
4.	760222832:55: +	TRINITY_DN35027_c3_g2_i12 was annotated as putative rRNA methyltransferase
5.	785047004:88: +	TRINITY_DN34404_c4_g1_i14 an auxin response factor
6.	633271872:58: +	Genic SNP: i*n mRNA of pin like transcript variants*
7.	593701379:271: +	TRINITY_DN32710_c2_g1_i2 annotated as F-box protein SKIP23
8.	376936577:87: −	TRINITY_DN34296_c0_g1_i10 a serine/threonine protein phosphatase 2A
9.	834373094:36: −	GENIC SNP: ribosomal protein S2
10.	834384838:29: −	GENIC SNP: cytochrome P450 b559 alpha subunit
11.	164755426:80: −	TRINITY_DN34186_c2_g3_i4; annotated as Cytochrome P450 89A2
12.	35373484:284: +	TRINITY_DN33874_c0_g1_i3 annotated as U-box domain-containing protein and TRINITY_DN34453_c0_g3_i10 annotated as chromatin structure remodelling complex protein BSH

Similarly, GWAS on PC2 in 2017–18 gave a total of 6 non redundant SNPs, though no association was found in PC2 2018–19 data (Table S13). As PC2 reflects the loading of SPP, these loci could be possibly regulating SPP traits. As no marker trait association was found with SPP trait in our analysis and also there is no consistency in PC2 based GWAS, these 6 loci were excluded from the annotation analysis.

### Comparison between the association mapping models

While association mapping make use of historical recombination to unravel marker trait association, it’s difficult to control false positives arising due to linkage disequilibrium (LD), family relatedness and population stratification^51,59^. As a result, choosing an appropriate association mapping model is critical for identifying true marker-trait associations and minimising both false positives and negatives. In essence, an ideal model must have a uniform distribution of expected and observed p-values. Thus, in this investigation, we examined the Q-Q plots generated by different models in order to identify actual causal maker trait relationships and best suited model. If a Q-Q plot has a straight line close to 1:1, it follows a uniform distribution, indicating that null hypothesis is true (no significant marker trait association is present), whereas deviation depicts the presence of association between testing markers and trait. Upward side deflation of lines represents a false positive association, while a false negative is represented by downward deflation. If the line is close to 1:1 ratio with a sharp upward deviated tail, it indicates that both false positives and false negatives were controlled and the presence of true associations can be inferred^60,61^. Usually most of the SNPs follow a uniform distribution as they are not in LD with a causal polymorphism, but the few that are in LD with a causal polymorphism will produce significant *p* values arising as ‘tail’.

**Figure 4:**
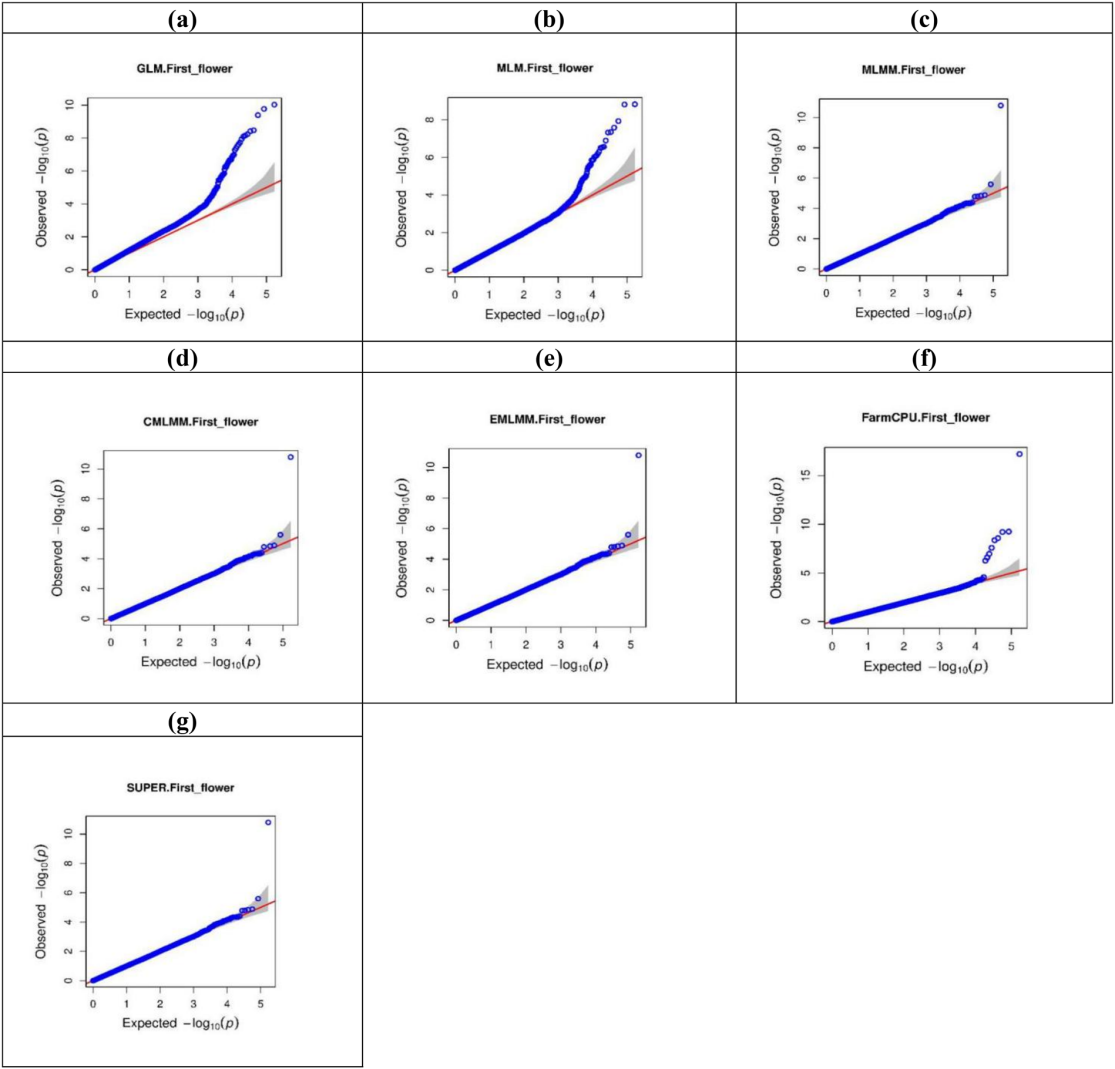
Quantile–Quantile (Q–Q) plots based on GWAS results from different association models for DOF in the year 2017–18. Model representations are GLM (**a**), MLM (**b**), MLMM (**c**), CMLM (**d**), ECMLM (**e**), FarmCPU (**f**) and SUPER (**g**). *x* axis plots expected − log_10_(*p*) values and *y* axis plots observed − log_10_(*p*) values respectively.

In our analysis, we compared seven models and found four models, viz*.* CMLM, ECMLM, MLMM, and SUPER, showed approximately 1:1 ratio, better than the remaining models, i.e*.*, GLM, MLM, and FarmCPU (Fig. 4 & S2–S10). When the MLM model is used in a genetically diverse panel, its superiority over GLM is lost as the random effect accounted for by the kinship matrix in the former is neutralised by the genetic diversity. Both GLM and MLM are single locus models, i.e. scanning one marker at a time, are computationally demanding and fail to decipher traits which are controlled by multiple loci. From Q-Q plots, it was evident that the GLM model was not able to remove the false positives arising due to LD, and therefore, all SNPs on scaffold NW_017988637.1 were found to be associated with both DOF and DFF (Fig. 3, S11–S14)^60,61^.

Though the multi-loci models, MLMM, CMLM and EMLM are more beneficial in mapping complex traits, they resort to overfitting and give rise to false negatives^49^. In the SUPER model, only the associated SNPs or pseudo quantitative trait nucleotides are used to derive kinship. MLM and its derivative models, which included kinship as covariates and perform overfitting of the data leading to the increased *p-*value threshold, were superior in controlling the false positives (Table S5-S13), but they also favoured false negatives. Hence, in most cases, these models were able to find only one SNP associated with both DOF and DFF (Fig. 3, S11–S14).

FarmCPU is superior over the other mapping models, as it incorporates multiple markers as covariates to remove confounding between the testing marker and kinship. In our analysis, it was found to be better than the other aforementioned models in dealing with both false positives and negative associations^60,61^. The FarmCPU model overcomes the limitation of false negatives due to overfitting (in the case of CMLM, EMLM, SUPER, and MLMM) and LD based false positive associations (in the case of GLM), as well as being a multi-loci based model, it was appropriate for dissecting the complex traits (Fig. 3 and S11–14).

### Methodological improvement and advantages of PC based GWAS

Even if we wish to analyse multiple traits, GWAS methodology is essentially based on “*single trait single variant association basis*”. However the phenotypes are not under control of a single locus and there is higher possibility that genetic variants can influence multiple traits or vice-versa. The resolution of understanding complex traits will increase if we study multiple traits simultaneously. PCA based GWAS is one such approach. It is a highly effective method for collecting information from highly correlated, complex and multiple traits through dimension reduction. Many studies have already been done which support the use of PC based GWAS for complex traits^18,62–64^. A comparison between the trait and PC based GWAS models suggested the use of PC based GWAS for efficient and high throughput deliverables. This strategy can decrease the likelihood of false positives by avoiding the multiple testing issues^65,66^. As the normal distribution of the phenotype is a must for performing GWAS, PC scores will lead over the single trait as PCA will transform the skewed original variables into an approximate normal distribution, producing reliable GWAS results^67^. Further, GWAS using PC scores may detect genomic regions that could be overlooked by using individual traits, since PC scores represent integrated variables^68^. As many genes contribute to the phenotype of multiple traits of complex architecture, it can be used to describe pleiotropy also. A PC based test has optimal power when the underlying multi-trait signal can be captured by the first PC, and otherwise it will have suboptimal performance^69^. In our analysis, we found some common associations as deciphered by both trait based and PC based GWAS, besides some novel associations identified by PC based GWAS owing to some critical genes during annotation, like locus 593701379:271: + identified exclusively by PC1 based studies was present in the vicinity of TRINITY_DN32710_c2_g1_i2 annotated as F-box protein SKIP23.

### Genomic prediction

**Figure 5:**
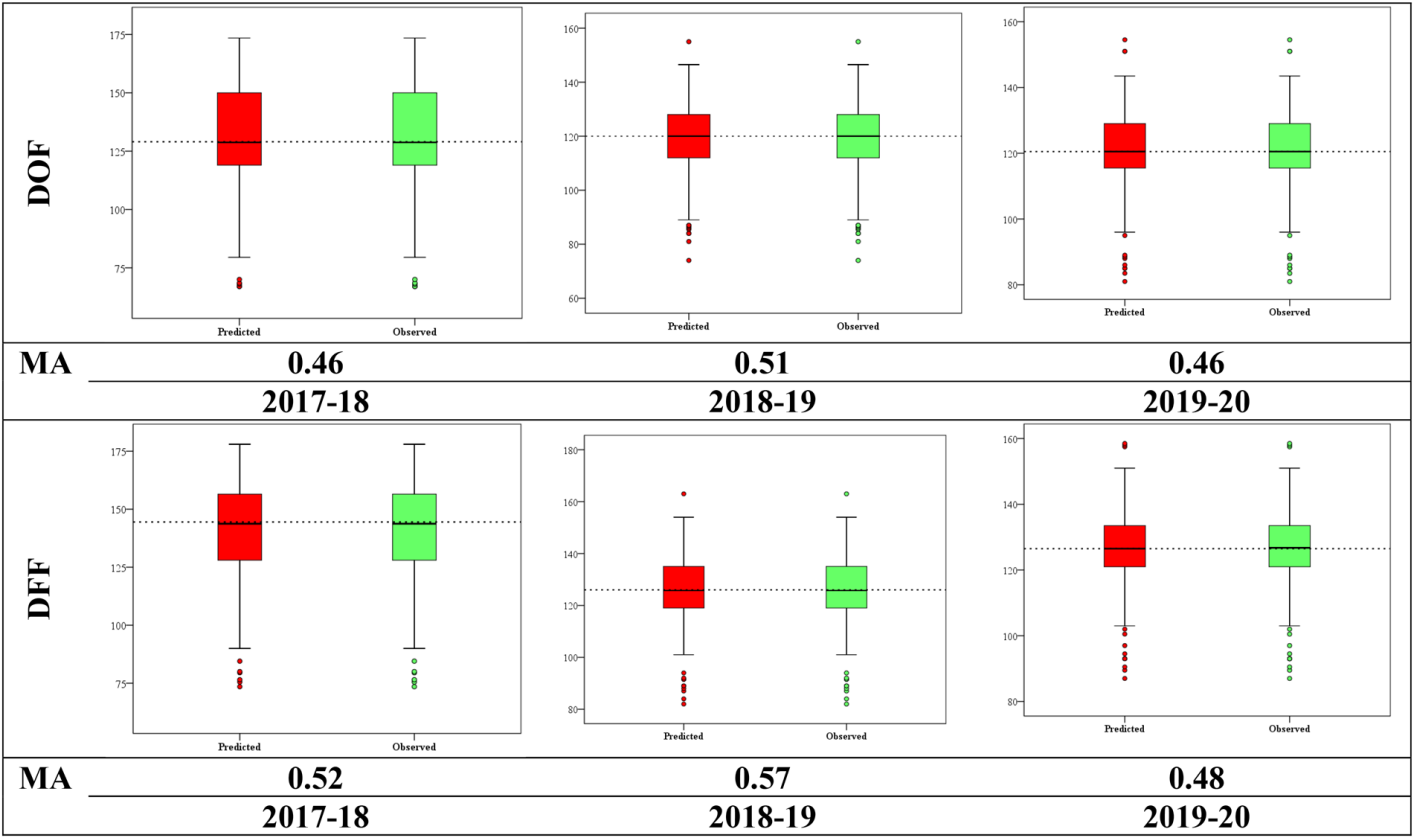
Box plot of observed traits vs. predicted flowering days through genomic prediction using RRBLUP method across different year’s data. The middle line in each box is the median value. Model accuracy (MA) is provided by setting 80:20 training and testing data sets.

The genomic estimated breeding value (GEBV) for each line was estimated using all the SNPs and 500 randomly generated train/test sets. The average correlation between the observed DOF and the predicted DOF by GP was 0.46, 0.51 and 0.46 in a model with no significant markers included as fixed effects during 2017–18, 2018–19, and 2019–20, respectively (Fig. 5). Similarly, the correlation between observed DFF and predicted DFF was 0.52, 0.57, and 0.48 during 2017–18, 2018–19 and 2019–20 respectively (Fig. 5). This is comparable to the prediction accuracies (PA) obtained for similar highly heritable traits, days to heading and days to maturity in wheat using a large number of Mexican and Iranian landraces. We observed moderate prediction accuracy in our data. Crossa et al.^70^, found the correlation values for plant height and yield to be 0.87 and 0.49, respectively pertaining to the trait complexity. Though a comparatively smaller number of lines were used in our study, we could still achieve ~ 50% accuracy, probably owing to the use of the core set. In Brassica, a similar or higher PA were achieved for grain yield related traits^71^. GP will enable high throughput evaluation of germplasm to identify superior one which can then be included in crop breeding programs to perform GP-based progeny selection^70,72^. However, the accuracy of GP models in predicting GEBV in pigeonpea should be increased by including more lines and including more environments for phenotyping to achieve reliable prediction and utilisation of the model.

### Annotation analysis for the significantly associated SNPs showing marker trait association

**Figure 6:**
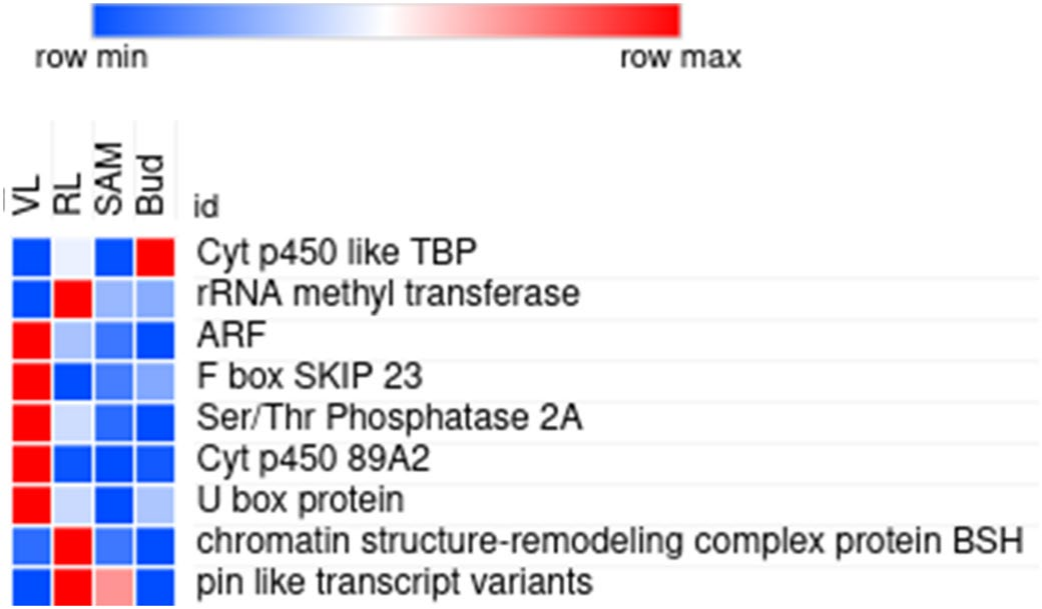
Expression pattern of the genes found in vicinity of associated SNPs which might have an important role in flowering. Vegetative leaves (VL), reproductive leaves (RL), shoot apical meristem (SAM) and reproductive buds (Bud).

All the 22 SNPs representing 12 different loci were considered for gene annotation analysis (Table 3). Four of the 12 loci were found to be present within or in the vicinity of vital flowering related genes (Table 4). The heatmap showing differential expression of nine of these genes in different tissues is presented in Fig. 6. The locus with three SNPs (812678863:261: +, 812679326:250: + and 812678807:41: +) present on the scaffold NW_017988637.1 revealed the presence of cytochrome P450-like TATA box binding protein (cytochrome P450-like TBP) within its vicinity. Several researchers have previously demonstrated the role of plant cytochrome P450s gene family members in various pathways, including hormone biosynthesis^73,74^, which have a bearing on both DOF and DFF, and this observation was strengthened by the expression data with the highest expression in floral bud tissues (Fig. 6).

SNP 785047004:88: + was located adjacent to the Auxin response factor (ARF), which is suggestive of its involvement in SAM to bud transition. Under low auxin concentration, AUX/IAA binds to ARFs, ultimately inhibiting the further downstream genes, whereas when present in higher concentrations, auxin binds to TIR1 (F box protein which is a component of E3 ubiquitin ligase). After auxin binds to TIR1, it gets activated and cleaves AUX/IAA, thereby freeing ARFs and ultimately leading to expression of auxin responsive genes^75–77^. In our analysis, the ARF gene was found to be express in VL, which is the phase where auxin is required for the transition from meristem to bud (Fig. 6).

Establishment of a high concentrations of auxin is required for floral induction^78–80^, which is generated by polar auxin transport involving regulators such as the auxin efflux carrier PIN-FORMED1 (PIN1) and the PINOID (PID) kinase, which controls PIN1 activity^81,82^. Interestingly, another SNP (633271872:58: +) was present in the genic region of PIN like transcript variants, which is reported to mediate auxin efflux dependent developmental processes as mutants of these showed defective auxin transport^82,83^. Also, PIN is believed to regulate flowering timing by altering auxin activity in collaboration with ARF. Likewise, a F box protein SKIP23 is a must for induction of downstream auxin responsive genes^84^ and was found in the vicinity of SNP (593701379:271: +). As it was found to be highly expressed in VL, it is hypothesised to degrade AUX/IAA and release ARF (found near other SNP i.e. 785047004:88: +).

Similarly, SNP (376936577:87: −) was very close to a serine/threonine protein phosphatase 2A. Although serine/threonine protein phosphatase 2A is known to participate in various stress signals^85^, few reports suggest its role in auxin as well as abscisic acid signalling^86,87^. Although it is not clear how this gene influences flowering, we presume that it regulates flowering by hormonal regulation, mainly by regulating ABA and auxin. Another SNP, 164755426:80: + was found near the cytochrome P450 89A2 subunit. CYP715 (a cyt p450 gene family member) appears to function as a key regulator of flower maturation, synchronising petal expansion and volatile emission^88^. Similarly, this SNP might be involved in the maturation of reproductive buds to flowers.

A transcript (TRINITY_DN33874_c0_g1_i3) present besides SNP (35373484:284: +) was annotated as a U-box domain-containing protein. The SPIN1 (SPL11-INteracting protein 1) gene has been reported to regulate flowering time in rice and it is ubiquitinated by SPL11 (a U box protein)^89^. The spl11 rice mutants were found to display delayed flowering under long-day conditions. As per a previous report, mutating a U box protein in rice (SPL11) leads to delayed flowering. Transcript (TRINITY_DN33874_c0_g1_i3) might regulate flowering time as in rice, but interestingly its expression was higher in VL only suggesting that it may regulate flowering through a different mechanism from that of rice.

Another transcript, TRINITY_DN34453_c0_g3_i10 annotated as chromatin structure remodelling complex protein BSH was found in the vicinity of SNP (35373484:284: +). Several components of chromatin remodelling complexes are evolutionarily conserved in plants, such as the SWI3 subunits^90^, SNF5/BSH subunit^91^, the nuclear actin-related protein ARP4^92^, BRAHMA (BRM), or SPLAYED (SYD). Both of these latter proteins are ATPases of Arabidopsis SWI/SNF complexes and have been shown to participate in the control of flower development and flowering time^93–95^. Likewise, the SWI3B protein interacts with the flowering regulator FCA^90^. Several reports regarding the involvement of epigenetic mechanisms in flower induction regulation are available, and TRINITY_DN34453_c0_g3_i10 might play a similar role. Interestingly, SNP 760222832:55: + was found in the vicinity of RNA methyltransferase (TRINITY_DN35027_c3_g2_i12). Many epigenetic regulators have already been reported to regulate flowering timing^96,97^. TRINITY_DN35027_c3_g2_i12 was found to express constantly in VL, Mer and Bud but the expression increased in RL, suggesting its role in flowering induction.

## Conclusion

In the current study, PC based GWAS was found to be superior over trait based and multi-loci based models for DOF and DFF in Pigeonpea, analysed using 142 accessions and 168,540 SNPs. PC transformation of the traits revealed that PC1 captured 58% of the variation, while PC1 and PC2 cumulatively captured 85% of the variation, suggesting PC1 and PC2 were sufficient enough for GWAS in our datasets. Cumulatively, GWAS revealed the association of 22 SNPs with DOF, DFF or PC1, out of which 15 were solely identified by trait based GWAS, 3 by both trait based as well as PC based GWAS, and 4 SNPs were found to be associated only through PC based GWAS. The 4 SNPs found to be associated with PC1 might be pleiotropic as PC1 also represented PH besides DOF and DFF. One of these 4 SNPs is annotated as F box protein, which plays a vital role in auxin signalling during growth and development, so these 4 SNPs can be inferred as pleiotropic to DOF, DFF and PH. Many of the associated SNPs were in the vicinity of vital genes like Auxin responsive genes like ARF, F box protein, U box protein, PIN like transcripts, chromatin remodelers, RNA methyltransferase the homolog’s/ortholog’s, many of which have been previously reported to regulate floral transition in other plant species. A few uncharacterized genes were also found, which are novel and need further characterization in order to decipher their function and role. Associations found in the present study suggest a functional basis of the associations in the regulation of flowering, and hence these genes are excellent candidates for further validation through bi-parental analysis followed by mutagenesis, genome editing, and other approaches. In conclusion, PC based GWAS is effective in deciphering pleiotropy and complex traits over trait based GWAS. Furthermore, the study can be taken forward by combining the PCA and the Multiple Dimension Scaling method to handle both quantitative and qualitative phenotypes as inputs for association mapping models.

## Supplementary Information

Supplementary Information.

## Data Availability

All sequencing data used in the current research work are available at (https://www.ncbi.nlm.nih.gov/) and the SRA accession numbers to access them are provided in Table S1.
