# Supplementary material for: Single trait versus principal component based association analysis for flowering related traits in pigeonpea
Source: Sci Rep. 2022 Jun 21;12:10453. doi: 10.1038/s41598-022-14568-1 (PMC9211048; doi:10.1038/s41598-022-14568-1)
Supplement: Supplementary file 1 — Supplementary Information. [file 41598_2022_14568_MOESM1_ESM.docx]

**Table S1:** Summary of 142 lines in terms of biological status, species, region, and geographic origin

| **Sl. no.** | **Accessions** | **SRA number** | **Biological status** | **Biological name** | **Region** | **Geographic origin** |
| --- | --- | --- | --- | --- | --- | --- |
| 1 | ICP 995 | SRR5484712 | Breeding line | *Cajanus cajan (L.) Millsp.* | South Asia | India |
| 2 | ICP 9750 | SRR5484714 | Breeding line | *Cajanus cajan (L.) Millsp.* | South Asia | India |
| 3 | ICP 9691 | SRR5484715 | Breeding line | *Cajanus cajan (L.) Millsp.* | South Asia | India |
| 4 | ICP 9655 | SRR5484717 | Breeding line | *Cajanus cajan (L.) Millsp.* | South Asia | India |
| 5 | ICP 9414 | SRR5484722 | Landrace | *Cajanus cajan (L.) Millsp.* | South Asia | India |
| 6 | ICP 939 | SRR5484723 | Landrace | *Cajanus cajan (L.) Millsp.* | South Asia | India |
| 7 | ICP 9336 | SRR5484724 | Landrace | *Cajanus cajan (L.) Millsp.* | South Asia | India |
| 8 | ICP 9045 | SRR5484731 | Landrace | *Cajanus cajan (L.) Millsp.* | South Asia | India |
| 9 | ICP 8949 | SRR5484732 | Landrace | *Cajanus cajan (L.) Millsp.* | South Asia | India |
| 10 | ICP 8921 | SRR5484734 | Landrace | *Cajanus cajan (L.) Millsp.* | South Asia | India |
| 11 | ICP 8863 | SRR5484735 | Breeding line | *Cajanus cajan (L.) Millsp.* | South Asia | India |
| 12 | ICP 8860 | SRR5484736 | Breeding line | *Cajanus cajan (L.) Millsp.* | South Asia | India |
| 13 | ICP 8840 | SRR5484737 | Landrace | *Cajanus cajan (L.) Millsp.* | South Asia | India |
| 14 | ICP 8793 | SRR5484740 | Landrace | *Cajanus cajan (L.) Millsp.* | South Asia | India |
| 15 | ICP 8757 | SRR5484741 | Landrace | *Cajanus cajan (L.) Millsp.* | South Asia | India |
| 16 | ICP 8700 | SRR5484742 | Landrace | *Cajanus cajan (L.) Millsp.* | South Asia | India |
| 17 | ICP 8602 | SRR5484744 | Landrace | *Cajanus cajan (L.) Millsp.* | South Asia | India |
| 18 | ICP 8384 | SRR5484745 | Landrace | *Cajanus cajan (L.) Millsp.* | South Asia | India |
| 19 | ICP 8266 | SRR5484746 | Breeding line | *Cajanus cajan (L.) Millsp.* | South Asia | India |
| 20 | ICP 8255 | SRR5484747 | Breeding line | *Cajanus cajan (L.) Millsp.* | South Asia | India |
| 21 | ICP 8227 | SRR5484749 | Breeding line | *Cajanus cajan (L.) Millsp.* | South Asia | India |
| 22 | ICP 8152 | SRR5484752 | Landrace | *Cajanus cajan (L.) Millsp.* | South Asia | India |
| 23 | ICP 8012 | SRR5484755 | Landrace | *Cajanus cajan (L.) Millsp.* | South Asia | India |
| 24 | ICP 7869 | SRR5484760 | Landrace | *Cajanus cajan (L.) Millsp.* | South Asia | India |
| 25 | ICP 7803 | SRR5484763 | Landrace | *Cajanus cajan (L.) Millsp.* | South Asia | India |
| 26 | ICP 772 | SRR5484765 | Landrace | *Cajanus cajan (L.) Millsp.* | South Asia | India |
| 27 | ICP 7507 | SRR5484767 | Landrace | *Cajanus cajan (L.) Millsp.* | South Asia | India |
| 28 | ICP 7426 | SRR5484771 | Landrace | *Cajanus cajan (L.) Millsp.* | South Asia | India |
| 29 | ICP 7375 | SRR5484775 | Landrace | *Cajanus cajan (L.) Millsp.* | South Asia | India |
| 30 | ICP 7366 | SRR5484776 | Landrace | *Cajanus cajan (L.) Millsp.* | South Asia | India |
| 31 | ICP 7314 | SRR5484778 | Breeding line | *Cajanus cajan (L.) Millsp.* | South Asia | India |
| 32 | ICP 7260 | SRR5484781 | Breeding line | *Cajanus cajan (L.) Millsp.* | South Asia | India |
| 33 | ICP 7223 | SRR5484783 | Breeding line | *Cajanus cajan (L.) Millsp.* | South Asia | India |
| 34 | ICP 7221 | SRR5484784 | Breeding line | *Cajanus cajan (L.) Millsp.* | South Asia | India |
| 35 | ICP 7148 | SRR5484786 | Breeding line | *Cajanus cajan (L.) Millsp.* | South Asia | Sri Lanka |
| 36 | ICP 7076 | SRR5484788 | Landrace | *Cajanus cajan (L.) Millsp.* | South Asia | India |
| 37 | ICP 7057 | SRR5484789 | Landrace | *Cajanus cajan (L.) Millsp.* | South Asia | India |
| 38 | ICP 7 | SRR5484791 | Landrace | *Cajanus cajan (L.) Millsp.* | South Asia | India |
| 39 | ICP 6992 | SRR5484792 | Landrace | *Cajanus cajan (L.) Millsp.* | South Asia | India |
| 40 | ICP 6971 | SRR5484794 | Breeding line | *Cajanus cajan (L.) Millsp.* | South Asia | India |
| 41 | ICP 6929 | SRR5484795 | Breeding line | *Cajanus cajan (L.) Millsp.* | Meso America | Trinidad and Tobago |
| 42 | ICP 6859 | SRR5484798 | Landrace | *Cajanus cajan (L.) Millsp.* | South Asia | India |
| 43 | ICP 6845 | SRR5484799 | Landrace | *Cajanus cajan (L.) Millsp.* | South Asia | India |
| 44 | ICP 6815 | SRR5484800 | Landrace | *Cajanus cajan (L.) Millsp.* | South Asia | India |
| 45 | ICP 6739 | SRR5484801 | Landrace | *Cajanus cajan (L.) Millsp.* | South Asia | India |
| 46 | ICP 6668 | SRR5484802 | Landrace | *Cajanus cajan (L.) Millsp.* | South Asia | India |
| 47 | ICP 655 | SRR5484803 | Landrace | *Cajanus cajan (L.) Millsp.* | South Asia | India |
| 48 | ICP 6370 | SRR5484805 | Other | *Cajanus cajan (L.) Millsp.* | Unknown | Unknown |
| 49 | ICP 6128 | SRR5484807 | Breeding line | *Cajanus cajan (L.) Millsp.* | South Asia | Bangladesh |
| 50 | ICP 6123 | SRR5484808 | Breeding line | *Cajanus cajan (L.) Millsp.* | South Asia | India |
| 51 | ICP 6049 | SRR5484809 | Landrace | *Cajanus cajan (L.) Millsp.* | South Asia | India |
| 52 | ICP 5863 | SRR5484811 | Landrace | *Cajanus cajan (L.) Millsp.* | South Asia | India |
| 53 | ICP 5142 | SRR5484813 | Breeding line | *Cajanus cajan (L.) Millsp.* | South Asia | India |
| 54 | ICP 4715 | SRR5484817 | Breeding line | *Cajanus cajan (L.) Millsp.* | Sub Saharian Africa | Ghana |
| 55 | ICP 4575 | SRR5484818 | Breeding line | *Cajanus cajan (L.) Millsp.* | South Asia | India |
| 56 | ICP 4392 | SRR5484819 | Landrace | *Cajanus cajan (L.) Millsp.* | South Asia | India |
| 57 | ICP 4317 | SRR5484820 | Landrace | *Cajanus cajan (L.) Millsp.* | South Asia | India |
| 58 | ICP 4307 | SRR5484821 | Landrace | *Cajanus cajan (L.) Millsp.* | South Asia | India |
| 59 | ICP 4167 | SRR5484824 | Landrace | *Cajanus cajan (L.) Millsp.* | South Asia | India |
| 60 | ICP 4029 | SRR5484825 | Breeding line | *Cajanus cajan (L.) Millsp.* | South Asia | India |
| 61 | ICP 3576 | SRR5484828 | Landrace | *Cajanus cajan (L.) Millsp.* | South Asia | India |
| 62 | ICP 348 | SRR5484829 | Breeding line | *Cajanus cajan (L.) Millsp.* | South Asia | India |
| 63 | ICP 3451 | SRR5484830 | Landrace | *Cajanus cajan (L.) Millsp.* | South Asia | India |
| 64 | ICP 3049 | SRR5484831 | Landrace | *Cajanus cajan (L.) Millsp.* | South Asia | India |
| 65 | ICP 3046 | SRR5484832 | Landrace | *Cajanus cajan (L.) Millsp.* | South Asia | India |
| 66 | ICP 2698 | SRR5484834 | Landrace | *Cajanus cajan (L.) Millsp.* | South Asia | India |
| 67 | ICP 2577 | SRR5484835 | Breeding line | *Cajanus cajan (L.) Millsp.* | South Asia | Myanmar |
| 68 | ICP 16309 | SRR5484843 | Breeding line | *Cajanus cajan (L.) Millsp.* | South Asia | India |
| 69 | ICP 16264 | SRR5484844 | Breeding line | *Cajanus cajan (L.) Millsp.* | South Asia | India |
| 70 | ICP 15493 | SRR5484856 | Landrace | *Cajanus cajan (L.) Millsp.* | Sub Saharian Africa | Uganda |
| 71 | ICP 15382 | SRR5484857 | Landrace | *Cajanus cajan (L.) Millsp.* | Sub Saharian Africa | Nigeria |
| 72 | ICP 15185 | SRR5484859 | Breeding line | *Cajanus cajan (L.) Millsp.* | South Asia | India |
| 73 | ICP 15161 | SRR5484861 | Breeding line | *Cajanus cajan (L.) Millsp.* | South Asia | India |
| 74 | ICP 15109 | SRR5484864 | Landrace | *Cajanus cajan (L.) Millsp.* | Sub Saharian Africa | Uganda |
| 75 | ICP 15068 | SRR5484865 | Breeding line | *Cajanus cajan (L.) Millsp.* | South Asia | India |
| 76 | ICP 14976 | SRR5484866 | Breeding line | *Cajanus cajan (L.) Millsp.* | South Asia | India |
| 77 | ICP 14903 | SRR5484871 | Breeding line | *Cajanus cajan (L.) Millsp.* | South Asia | India |
| 78 | ICP 14900 | SRR5484872 | Breeding line | *Cajanus cajan (L.) Millsp.* | South Asia | India |
| 79 | ICP 14832 | SRR5484876 | Breeding line | *Cajanus cajan (L.) Millsp.* | South Asia | India |
| 80 | ICP 14819 | SRR5484877 | Breeding line | *Cajanus cajan (L.) Millsp.* | South Asia | India |
| 81 | ICP 14722 | SRR5484880 | Breeding line | *Cajanus cajan (L.) Millsp.* | South Asia | India |
| 82 | ICP 14701 | SRR5484881 | Breeding line | *Cajanus cajan (L.) Millsp.* | South Asia | India |
| 83 | ICP 14638 | SRR5484882 | Breeding line | *Cajanus cajan (L.) Millsp.* | South Asia | India |
| 84 | ICP 14569 | SRR5484884 | Landrace | *Cajanus cajan (L.) Millsp.* | South Asia | Thailand |
| 85 | ICP 14471 | SRR5484887 | Breeding line | *Cajanus cajan (L.) Millsp.* | South Asia | India |
| 86 | ICP 14444 | SRR5484889 | Breeding line | *Cajanus cajan (L.) Millsp.* | South Asia | India |
| 87 | ICP 14368 | SRR5484892 | Breeding line | *Cajanus cajan (L.) Millsp.* | South America | Venezuela |
| 88 | ICP 14294 | SRR5484894 | Breeding line | *Cajanus cajan (L.) Millsp.* | Europe | Italy |
| 89 | ICP 14229 | SRR5484895 | Landrace | *Cajanus cajan (L.) Millsp.* | Sub Saharian Africa | Zambia |
| 90 | ICP 14147 | SRR5484899 | Breeding line | *Cajanus cajan (L.) Millsp.* | South America | Brazil |
| 91 | ICP 14120 | SRR5484901 | Landrace | *Cajanus cajan (L.) Millsp.* | Meso America | Jamaica |
| 92 | ICP 14116 | SRR5484902 | Landrace | *Cajanus cajan (L.) Millsp.* | Meso America | Jamaica |
| 93 | ICP 14094 | SRR5484903 | Landrace | *Cajanus cajan (L.) Millsp.* | South America | Venezuela |
| 94 | ICP 13884 | SRR5484909 | Landrace | *Cajanus cajan (L.) Millsp.* | Meso America | Puerto Rico |
| 95 | ICP 13662 | SRR5484912 | Landrace | *Cajanus cajan (L.) Millsp.* | South Asia | India |
| 96 | ICP 13633 | SRR5484913 | Breeding line | *Cajanus cajan (L.) Millsp.* | Sub Saharian Africa | Nigeria |
| 97 | ICP 13579 | SRR5484914 | Landrace | *Cajanus cajan (L.) Millsp.* | South Asia | Philippines |
| 98 | ICP 13577 | SRR5484915 | Breeding line | *Cajanus cajan (L.) Millsp.* | East Asia | China |
| 99 | ICP 13575 | SRR5484916 | Landrace | *Cajanus cajan (L.) Millsp.* | Sub Saharian Africa | Sierra Leone |
| 100 | ICP 13571 | SRR5484917 | Breeding line | *Cajanus cajan (L.) Millsp.* | Sub Saharian Africa | Nigeria |
| 101 | ICP 13431 | SRR5484920 | Landrace | *Cajanus cajan (L.) Millsp.* | Sub Saharian Africa | Malawi |
| 102 | ICP 13359 | SRR5484921 | Landrace | *Cajanus cajan (L.) Millsp.* | Sub Saharian Africa | Malawi |
| 103 | ICP 13304 | SRR5484922 | Landrace | *Cajanus cajan (L.) Millsp.* | Europe | Italy |
| 104 | ICP 13270 | SRR5484923 | Landrace | *Cajanus cajan (L.) Millsp.* | Sub Saharian Africa | Kenya |
| 105 | ICP 13191 | SRR5484927 | Breeding line | *Cajanus cajan (L.) Millsp.* | Oceania-Pacific | Australia |
| 106 | ICP 13167 | SRR5484928 | Landrace | *Cajanus cajan (L.) Millsp.* | Sub Saharian Africa | Kenya |
| 107 | ICP 13139 | SRR5484929 | Landrace | *Cajanus cajan (L.) Millsp.* | Sub Saharian Africa | Kenya |
| 108 | ICP 13011 | SRR5484931 | Landrace | *Cajanus cajan (L.) Millsp.* | South Asia | India |
| 109 | ICP 1279 | SRR5484936 | Landrace | *Cajanus cajan (L.) Millsp.* | South Asia | India |
| 110 | ICP 1273 | SRR5484939 | Landrace | *Cajanus cajan (L.) Millsp.* | South Asia | India |
| 111 | ICP 12680 | SRR5484940 | Breeding line | *Cajanus cajan (L.) Millsp.* | South Asia | India |
| 112 | ICP 12654 | SRR5484941 | Breeding line | *Cajanus cajan (L.) Millsp.* | South Asia | India |
| 113 | ICP 12515 | SRR5484943 | Landrace | *Cajanus cajan (L.) Millsp.* | South Asia | India |
| 114 | ICP 12410 | SRR5484944 | Landrace | *Cajanus cajan (L.) Millsp.* | South Asia | India |
| 115 | ICP 12298 | SRR5484945 | Landrace | *Cajanus cajan (L.) Millsp.* | South Asia | India |
| 116 | ICP 12142 | SRR5484948 | Landrace | *Cajanus cajan (L.) Millsp.* | Sub Saharian Africa | Tanzania |
| 117 | ICP 12123 | SRR5484949 | Landrace | *Cajanus cajan (L.) Millsp.* | Sub Saharian Africa | Tanzania |
| 118 | ICP 12105 | SRR5484950 | Landrace | *Cajanus cajan (L.) Millsp.* | Sub Saharian Africa | Tanzania |
| 119 | ICP 11946 | SRR5484954 | Landrace | *Cajanus cajan (L.) Millsp.* | South Asia | India |
| 120 | ICP 11910 | SRR5484955 | Landrace | *Cajanus cajan (L.) Millsp.* | South Asia | India |
| 121 | ICP 11833 | SRR5484957 | Landrace | *Cajanus cajan (L.) Millsp.* | South Asia | India |
| 122 | ICP 11823 | SRR5484958 | Landrace | *Cajanus cajan (L.) Millsp.* | South Asia | India |
| 123 | ICP 11690 | SRR5484961 | Breeding line | *Cajanus cajan (L.) Millsp.* | South Asia | India |
| 124 | ICP 11627 | SRR5484963 | Breeding line | *Cajanus cajan (L.) Millsp.* | South Asia | India |
| 125 | ICP 1156 | SRR5484965 | Landrace | *Cajanus cajan (L.) Millsp.* | South Asia | India |
| 126 | ICP 11543 | SRR5484966 | Breeding line | *Cajanus cajan (L.) Millsp.* | South Asia | India |
| 127 | ICP 11477 | SRR5484968 | Other | *Cajanus cajan (L.) Millsp.* | Unknown | Unknown |
| 128 | ICP 11320 | SRR5484973 | Landrace | *Cajanus cajan (L.) Millsp.* | South Asia | Nepal |
| 129 | ICP 11281 | SRR5484974 | Breeding line | *Cajanus cajan (L.) Millsp.* | South Asia | India |
| 130 | ICP 1126 | SRR5484975 | Breeding line | *Cajanus cajan (L.) Millsp.* | South Asia | India |
| 131 | ICP 11230 | SRR5484978 | Breeding line | *Cajanus cajan (L.) Millsp.* | South Asia | India |
| 132 | ICP 11059 | SRR5484982 | Breeding line | *Cajanus cajan (L.) Millsp.* | South Asia | India |
| 133 | ICP 11015 | SRR5484983 | Breeding line | *Cajanus cajan (L.) Millsp.* | South Asia | India |
| 134 | ICP 1071 | SRR5484989 | Breeding line | *Cajanus cajan (L.) Millsp.* | South Asia | India |
| 135 | ICP 10654 | SRR5484991 | Breeding line | *Cajanus cajan (L.) Millsp.* | South Asia | India |
| 136 | ICP 10559 | SRR5484993 | Landrace | *Cajanus cajan (L.) Millsp.* | South Asia | India |
| 137 | ICP 10503 | SRR5484996 | Landrace | *Cajanus cajan (L.) Millsp.* | South Asia | India |
| 138 | ICP 10447 | SRR5484997 | Landrace | *Cajanus cajan (L.) Millsp.* | South Asia | India |
| 139 | ICP 10397 | SRR5484998 | Landrace | *Cajanus cajan (L.) Millsp.* | South Asia | India |
| 140 | ICP 10228 | SRR5485002 | Landrace | *Cajanus cajan (L.) Millsp.* | South Asia | India |
| 141 | ICP 10094 | SRR5485003 | Landrace | *Cajanus cajan (L.) Millsp.* | South Asia | India |
| 142 | ICP 11321 | SRR5501032 | Landrace | *Cajanus cajan (L.) Millsp.* | South Asia | Nepal |

**Table S2:** Summary of principal component analysis result

| **Year** | **2017-18** | | | | | | | **2018-19** | | | | | | |
| --- | --- | --- | --- | --- | --- | --- | --- | --- | --- | --- | --- | --- | --- | --- |
| **PCs** | **Eigen values** | | **%Variance** | | **%Cumulative variance** | | | **Eigen values** | | **%Variance** | | **%Cumulative Variance** | | |
| PC1 | 2.36 | | 58.92 | | 58.92 | | | 2.30 | | 57.41 | | 57.41 | | |
| PC2 | 1.04 | | 26.08 | | 85.00 | | | 1.03 | | 25.70 | | 83.11 | | |
| PC3 | 0.55 | | 13.70 | | 98.69 | | | 0.66 | | 16.62 | | 99.74 | | |
| PC4 | 0.05 | | 1.31 | | 100.00 | | | 0.01 | | 0.26 | | 100.00 | | |
| **Traits** | | **DFF** | | **DOF** | | **PH** | **SPP** | **DFF** | **DOF** | | **PH** | | **SPP** |  |
| PC1 | | 0.63 | | 0.62 | | 0.48 | 0.06 | 0.64 | 0.63 | | 0.42 | | 0.11 |  |
| PC2 | | 0.08 | | 0.05 | | -0.30 | 0.95 | 0.03 | 0.04 | | -0.36 | | 0.94 |  |
| PC3 | | -0.32 | | -0.34 | | 0.83 | 0.31 | -0.30 | -0.31 | | 0.84 | | 0.34 |  |
| PC4 | | -0.71 | | 0.71 | | 0.00 | 0.02 | 0.71 | -0.71 | | -0.01 | | 0.01 |  |

**Table S3:** Cumulative descriptive statistics for the phenotypic traits for 2017-18 and 2018-19

| 1. **No.** | **Variable** | **Min.** | **Max.** | **Mean** | **Standard Deviation** | **Coefficient of Variation %** |
| --- | --- | --- | --- | --- | --- | --- |
| 1. | DOF | 67 | 174 | 125.25 | 0.83 | 15.83 |
| 2. | DFF | 73 | 178 | 133.14 | 0.85 | 15.39 |
| 3. | PH | 118.5 | 261 | 208.02 | 1.01 | 11.64 |
| 4. | SPP | 2.1 | 5.8 | 3.76 | 0.02 | 16.62 |

**Table S4:** Correlation analysis between the trait analysed (cumulatively between 2017-18 and 2018-19 data)

|  | **DOF** | **DFF** | **PH** | **SPP** |
| --- | --- | --- | --- | --- |
| **DOF** | 1 | 0.95 | 0.50 | 0.11 |
| **DFF** | 0.95 | 1 | 0.50 | 0.10 |
| **PH** | 0.50 | 0.50 | 1 | -0.05 |
| **SPP** | 0.11 | 0.10 | -0.05 | 1 |


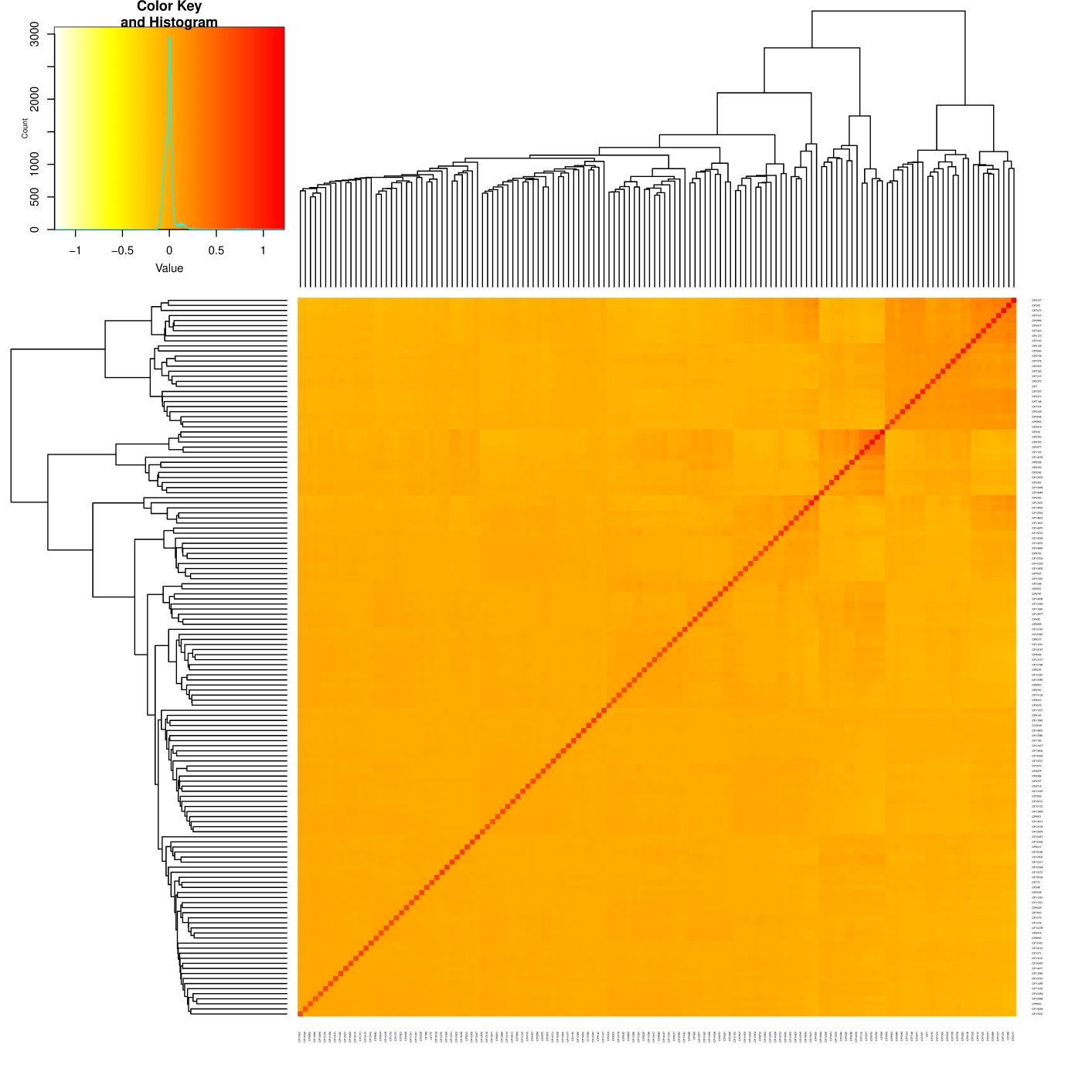


**Figure S1:** Heatmap and dendrogram of kinship matrix estimated using 168541 SNPs.

| **(a)** | **(b)** | **(c)** |
| --- | --- | --- |
| 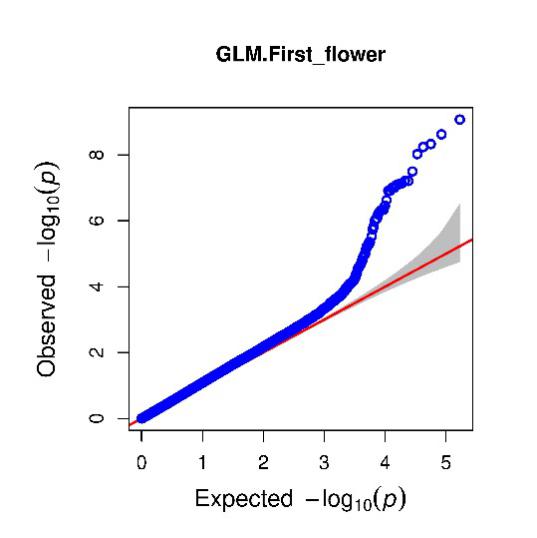 | 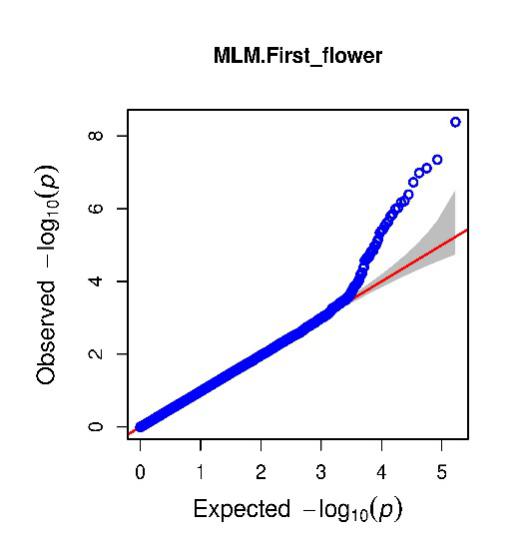 | 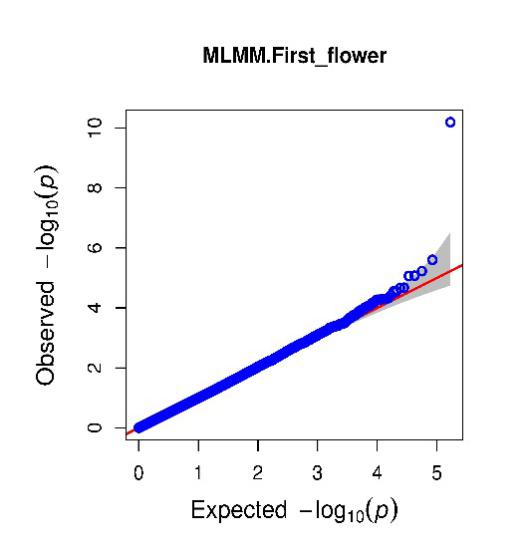 |
| **(d)** | **(e)** | **(f)** |
| 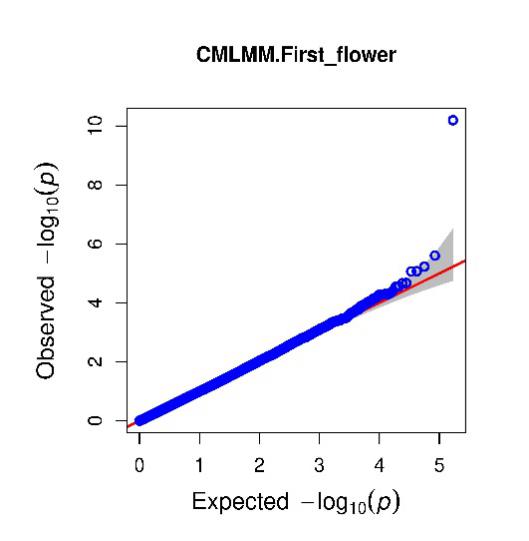 | 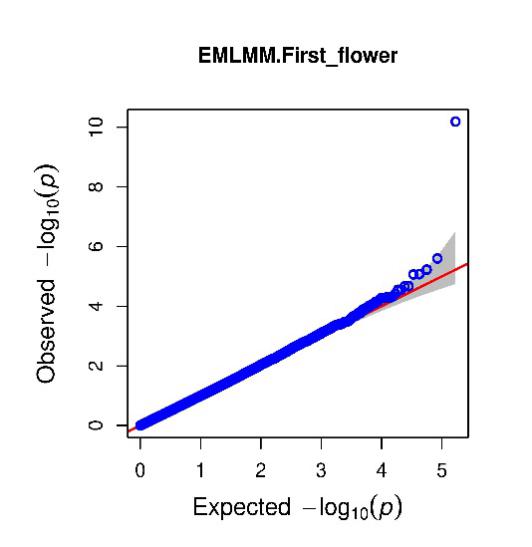 | 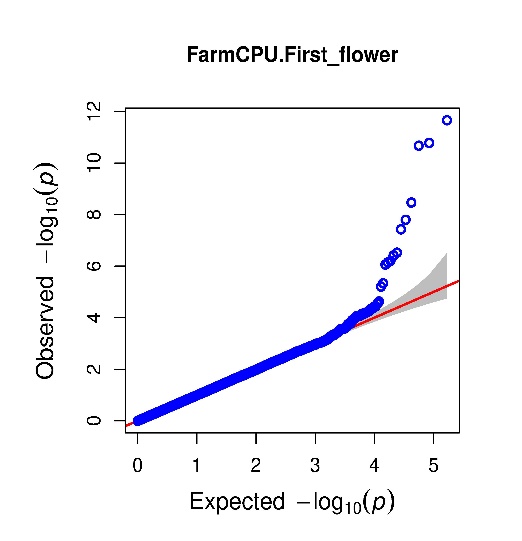 |
| **(g)** | **Figure S2:** Quantile-Quantile (Q-Q) plots based on GWAS results from different association models for DOF in the year 2018-19. Model representations are GLM (a), MLM (b), MLMM (c), CMLM(d), ECMLM(e), FarmCPU(f) and SUPER(g). *x* axis plots expected –log_10_(*p*) values and *y* axis plots observed –log_10_(*p*) values respectively. | |
| 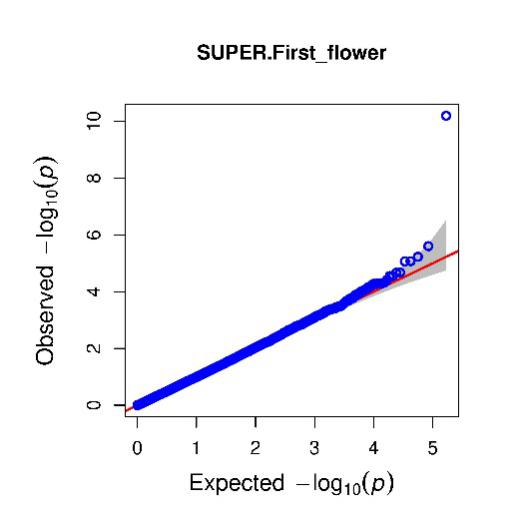 |  |  |

| **(a)** | **(b)** | **(c)** |
| --- | --- | --- |
| 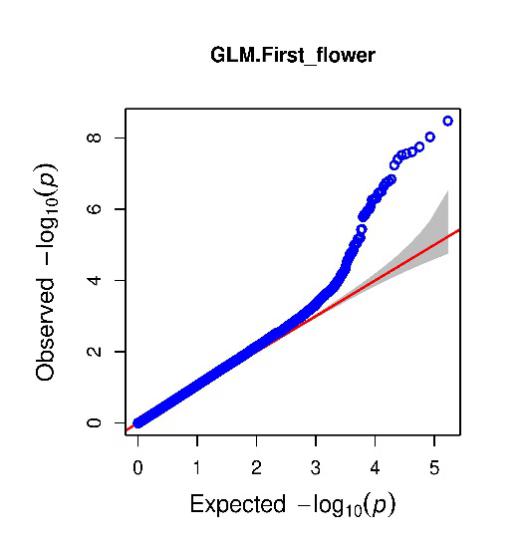 | 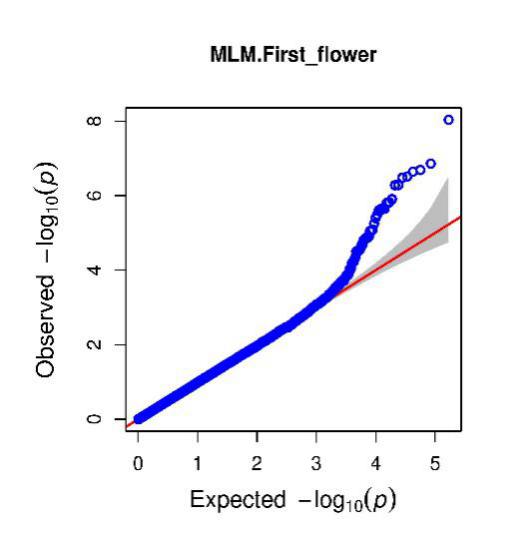 | 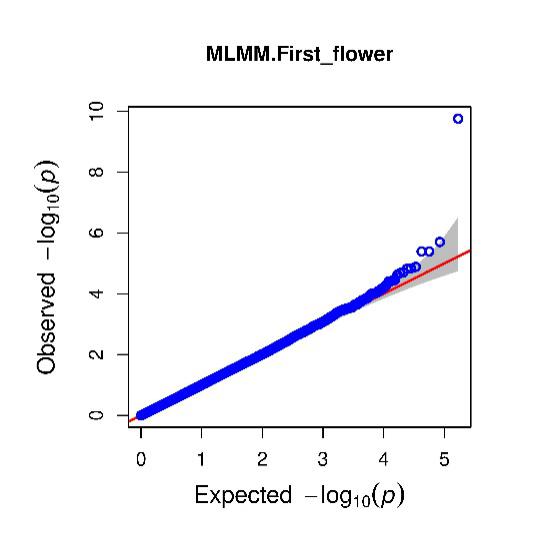 |
| **(d)** | **(e)** | **(f)** |
| 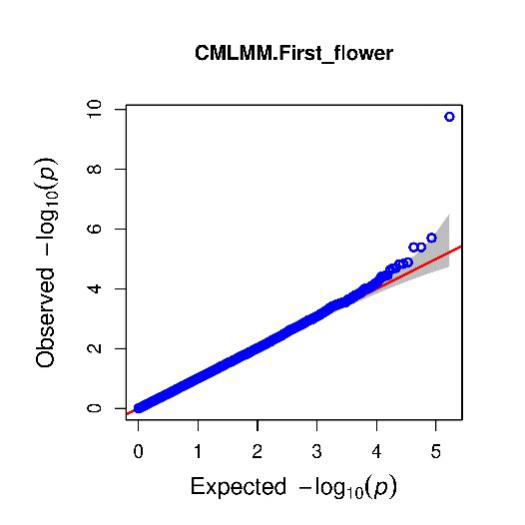 | 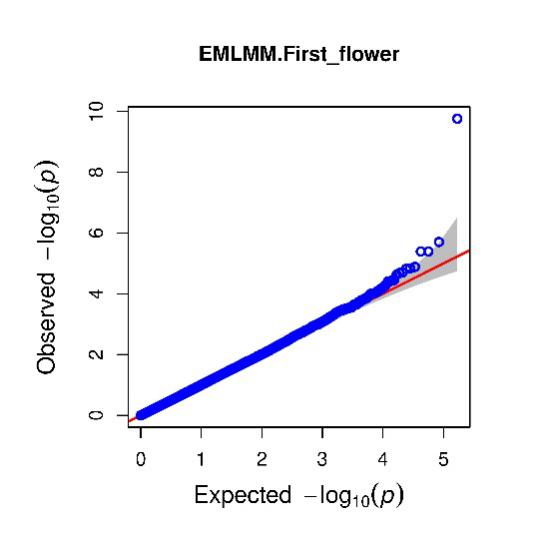 | 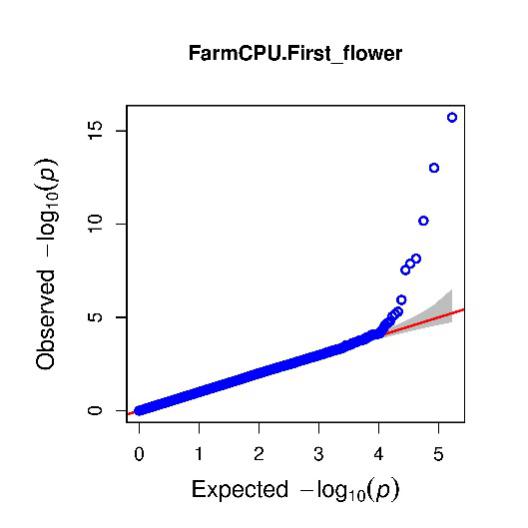 |
| **(g)** | **Figure S3:** Quantile-Quantile (Q-Q) plots based on GWAS results from different association models for DOF in the year 2019-20. Model representations are GLM (a), MLM (b), MLMM (c), CMLM(d), ECMLM(e), FarmCPU(f) and SUPER(g). *x* axis plots expected –log_10_(*p*) values and *y* axis plots observed –log_10_(*p*) values respectively. | |
| 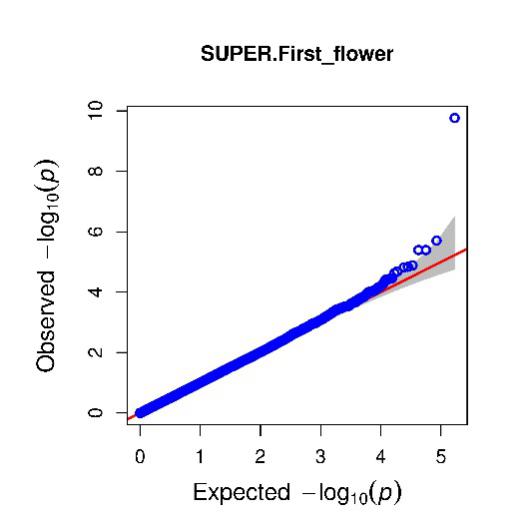 |  |  |

| **(a)** | **(b)** | **(c)** |
| --- | --- | --- |
| 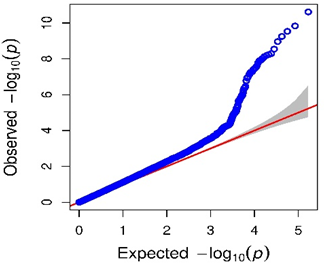 | 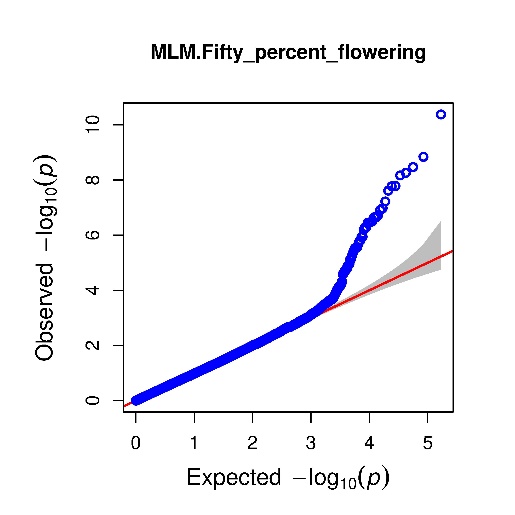 | 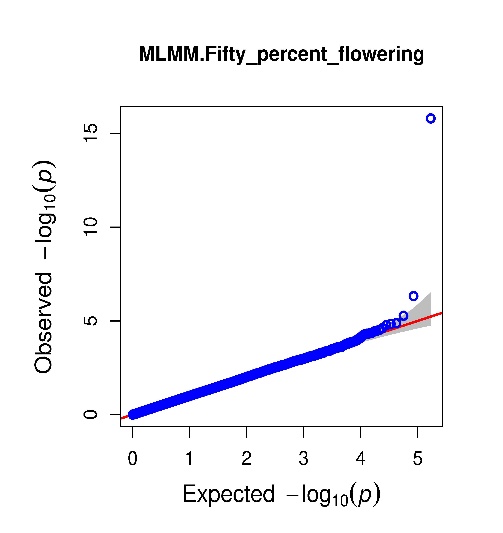 |
| **(d)** | **(e)** | **(f)** |
| 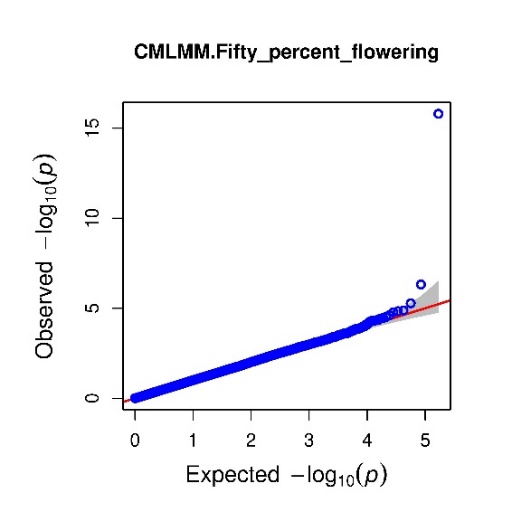 | 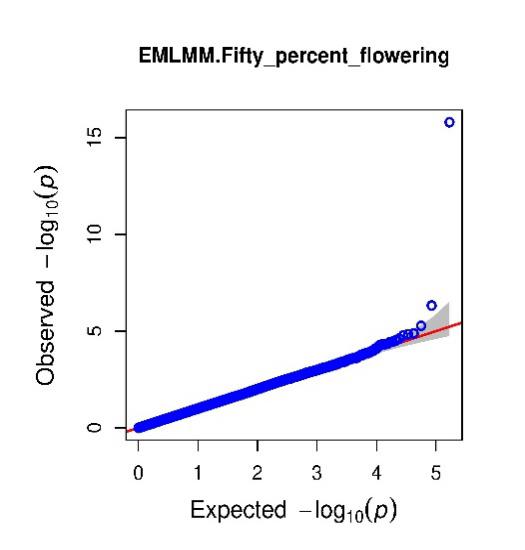 | 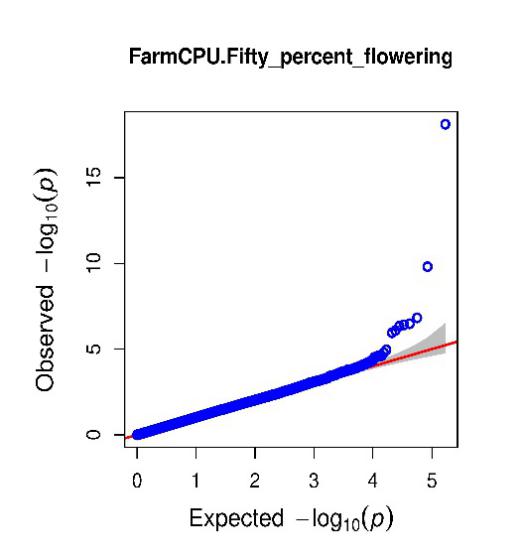 |
| **(g)** | **Figure S4:** Quantile-Quantile (Q-Q) plots based on GWAS results from different association models for DFF in the year 2017-18. Model representations are GLM (a), MLM (b), MLMM (c), CMLM(d), ECMLM(e), FarmCPU(f) and SUPER(g). *x* axis plots expected –log_10_(*p*) values and *y* axis plots observed –log_10_(*p*) values respectively. | |
| 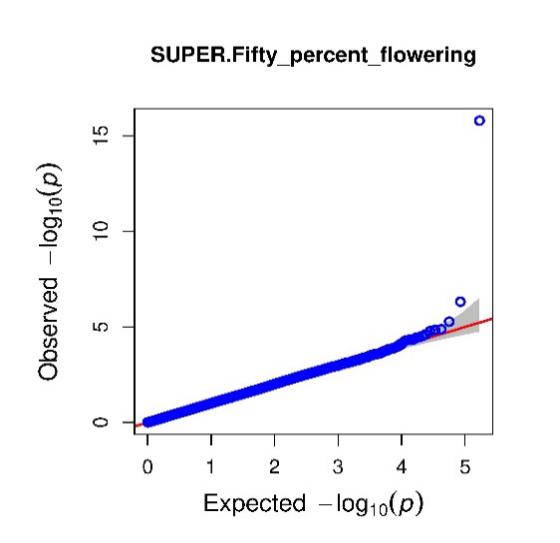 |  |  |

| **(a)** | **(b)** | **(c)** |
| --- | --- | --- |
| 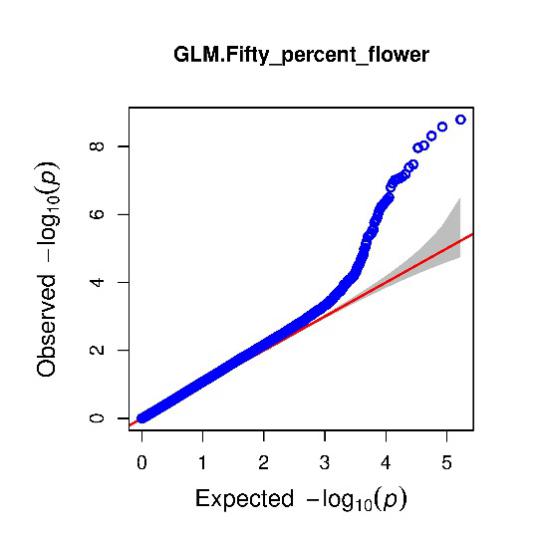 | 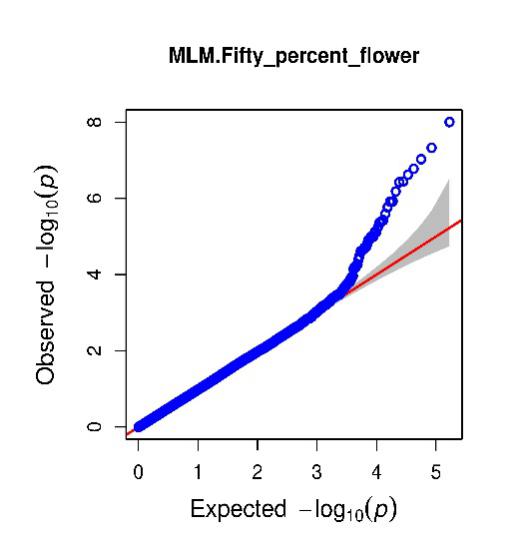 | 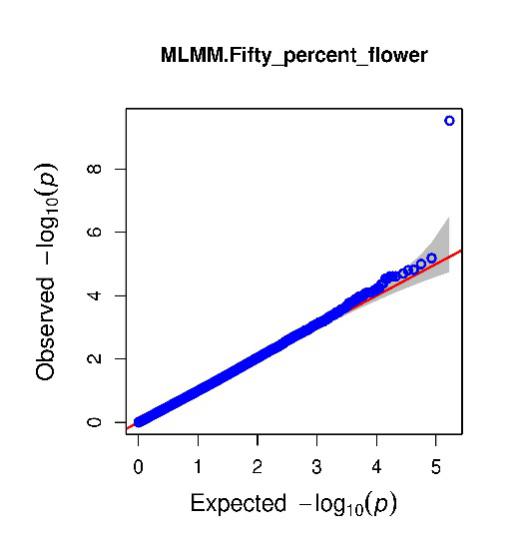 |
| **(d)** | **(e)** | **(f)** |
| 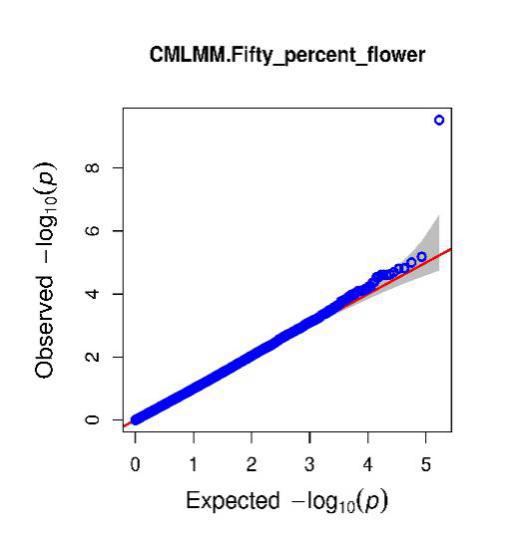 | 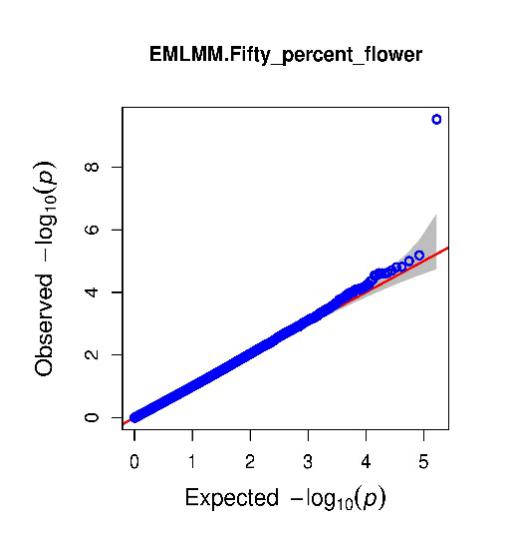 | 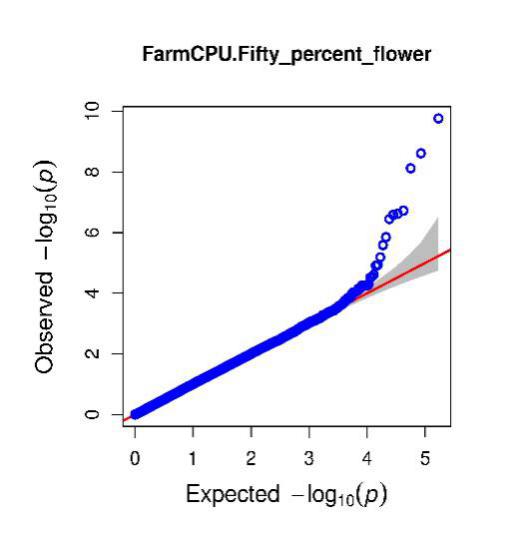 |
| **(g)** | **Figure S5:** Quantile-Quantile (Q-Q) plots based on GWAS results from different association models for DFF in the year 2018-19. Model representations are GLM (a), MLM (b), MLMM (c), CMLM(d), ECMLM(e), FarmCPU(f) and SUPER(g). *x* axis plots expected –log_10_(*p*) values and *y* axis plots observed –log_10_(*p*) values respectively. | |
| 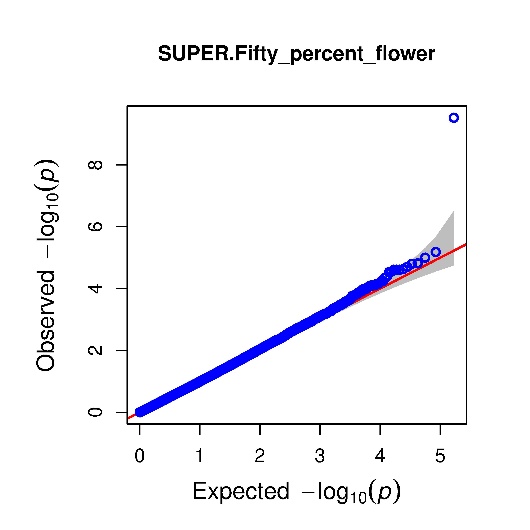 |  |  |

| **(a)** | **(b)** | **(c)** |
| --- | --- | --- |
| 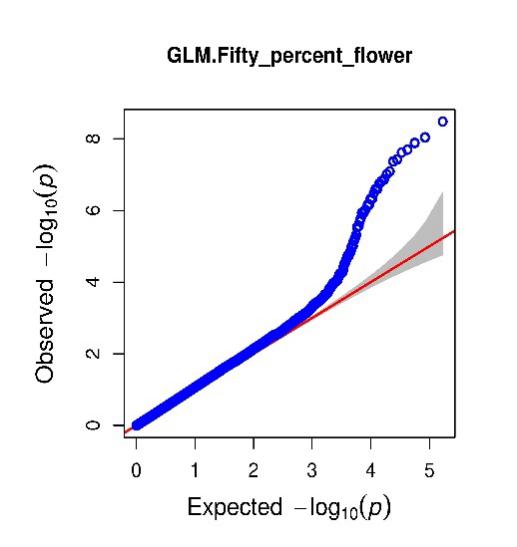 | 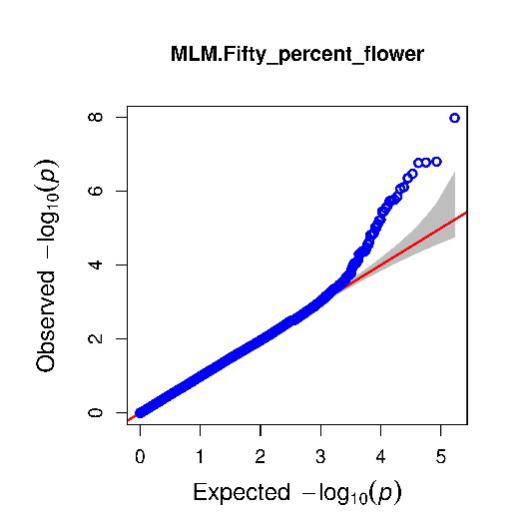 | 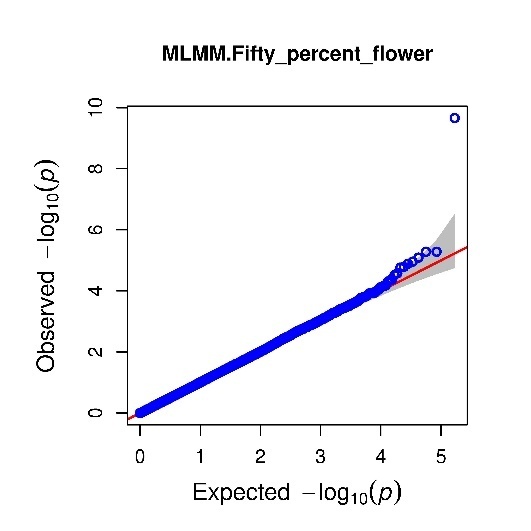 |
| **(d)** | **(e)** | **(f)** |
| 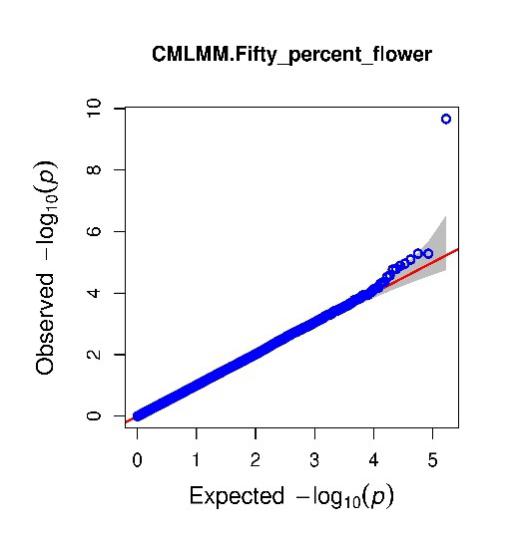 | 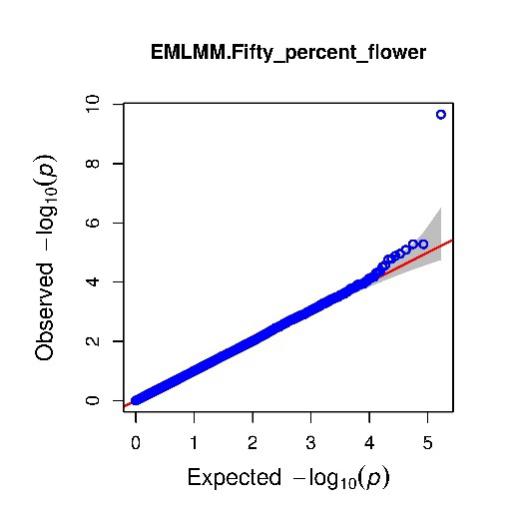 | 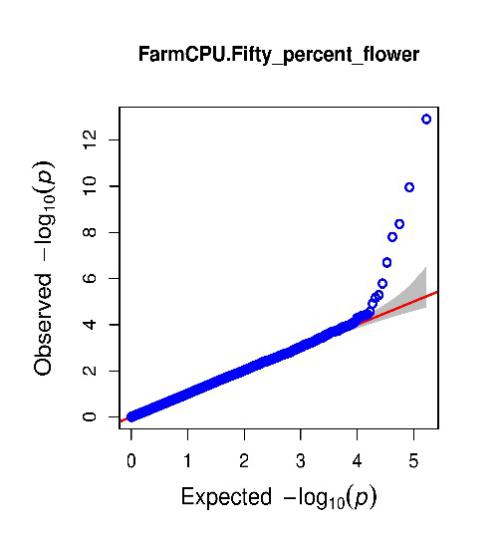 |
| **(g)** | **Figure S6:** Quantile-Quantile (Q-Q) plots based on GWAS results from different association models for DFF in the year 2019-20. Model representations are GLM (a), MLM (b), MLMM (c), CMLM(d), ECMLM(e), FarmCPU(f) and SUPER(g). *x* axis plots expected –log_10_(*p*) values and *y* axis plots observed –log_10_(*p*) values respectively. | |
| 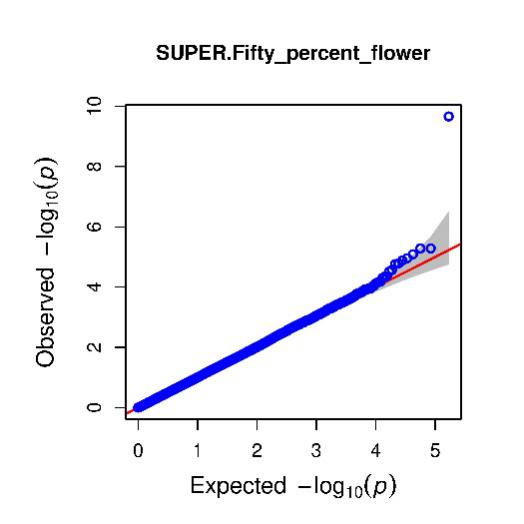 |  |  |

| **(a)** | **(b)** | **(c)** |
| --- | --- | --- |
| 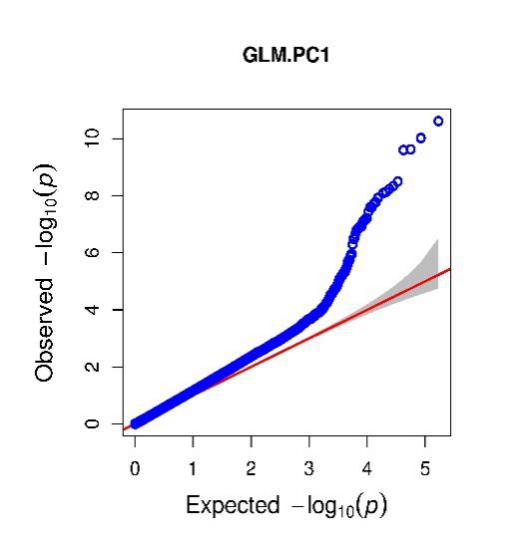 | 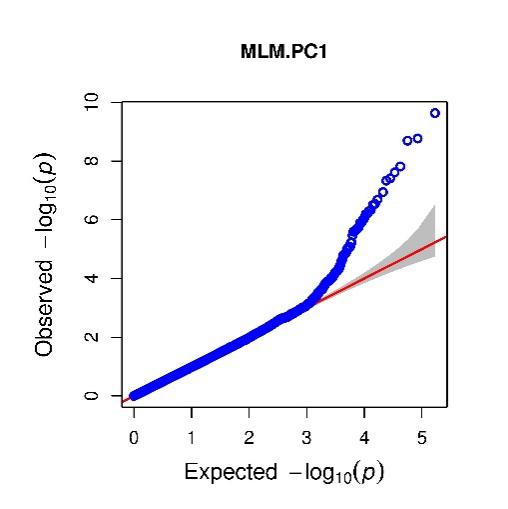 | 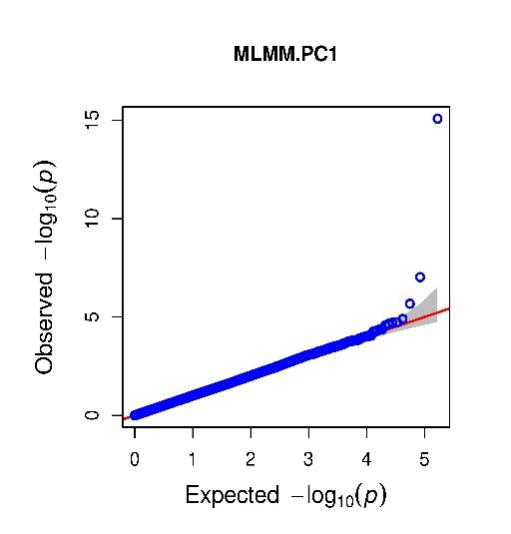 |
| **(d)** | **(e)** | **(f)** |
| 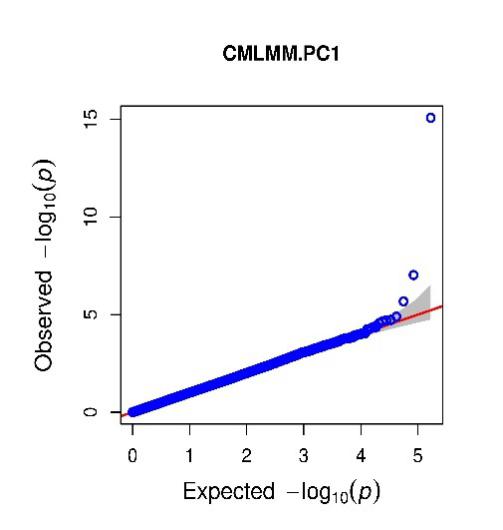 | 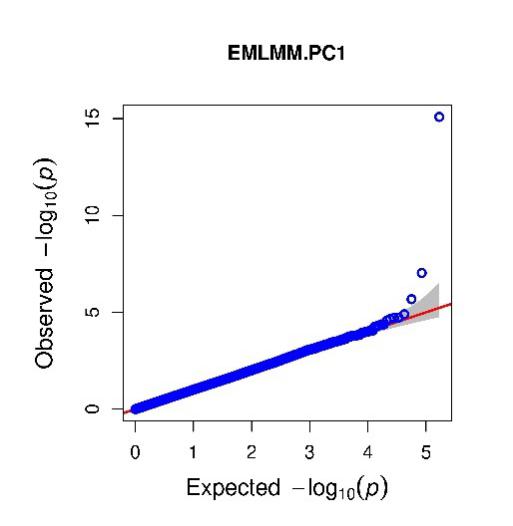 | 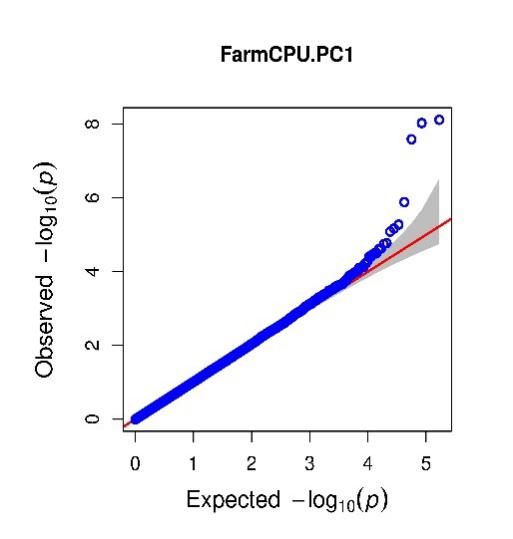 |
| **(g)** | **Figure S7:** Quantile-Quantile (Q-Q) plots based on GWAS results from different association models for PC1 in the year 2017-18. Model representations are GLM (a), MLM (b), MLMM (c), CMLM(d), ECMLM(e), FarmCPU(f) and SUPER(g). *x* axis plots expected –log_10_(*p*) values and *y* axis plots observed –log_10_(*p*) values respectively. | |
| 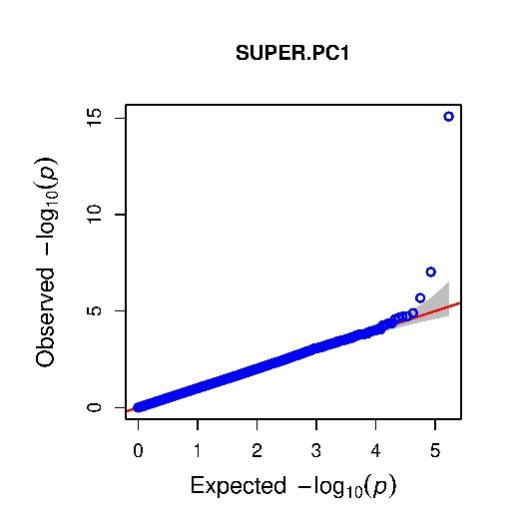 |  |  |

| **(a)** | **(b)** | **(c)** |
| --- | --- | --- |
| 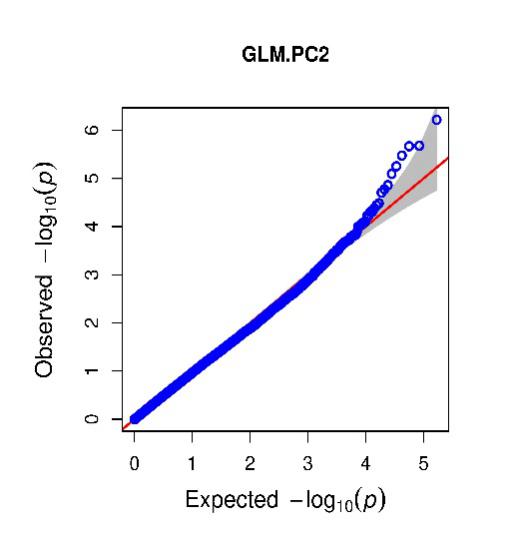 | 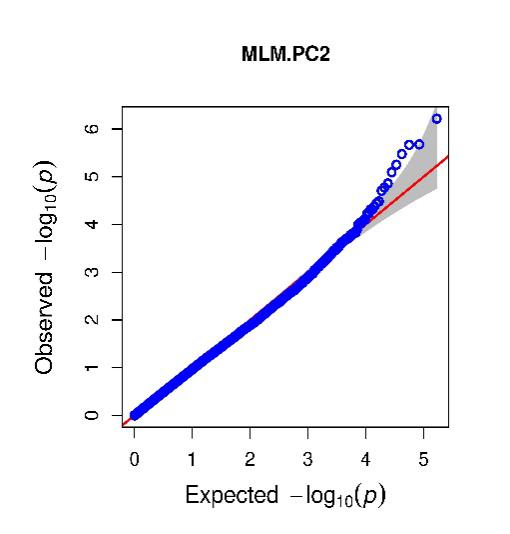 | 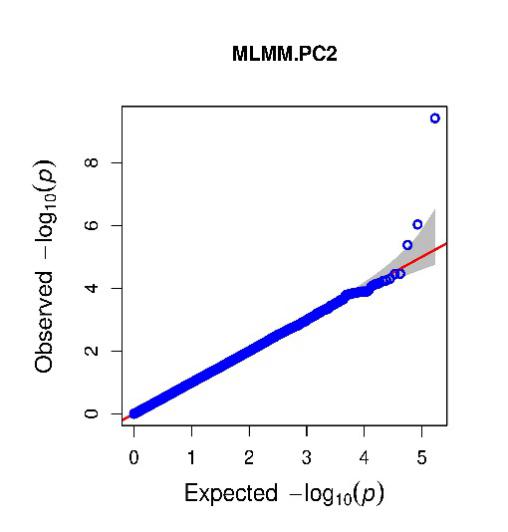 |
| **(d)** | **(e)** | **(f)** |
| 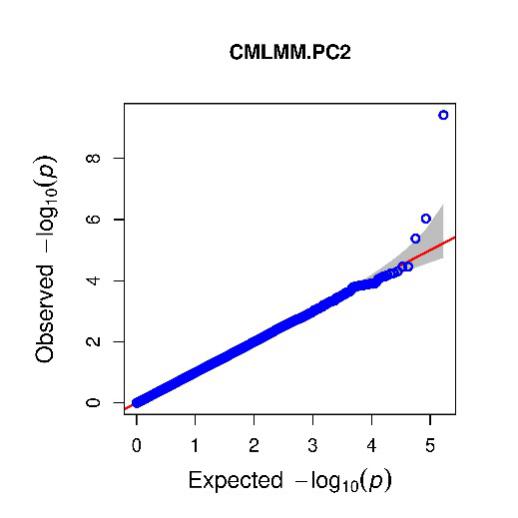 | 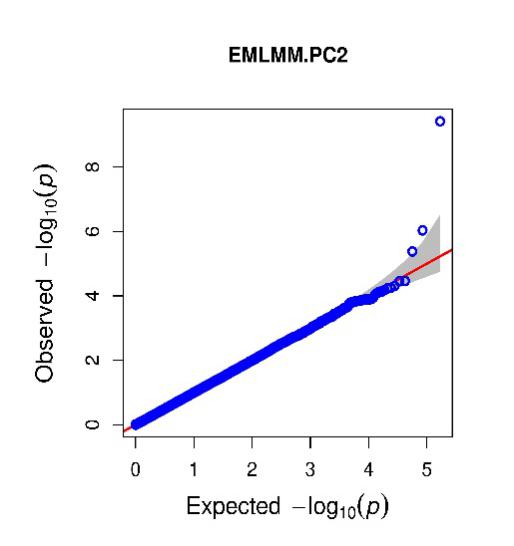 | 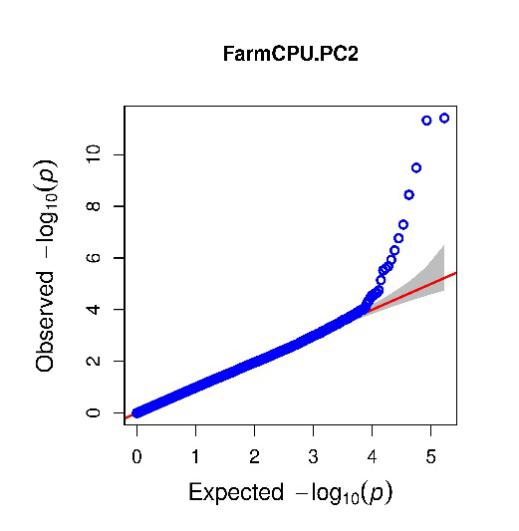 |
| **(g)** | **Figure S8:** Quantile-Quantile (Q-Q) plots based on GWAS results from different association models for PC2 in the year 2017-18. Model representations are GLM (a), MLM (b), MLMM (c), CMLM(d), ECMLM(e), FarmCPU(f) and SUPER(g). *x* axis plots expected –log_10_(*p*) values and *y* axis plots observed –log_10_(*p*) values respectively. | |
| 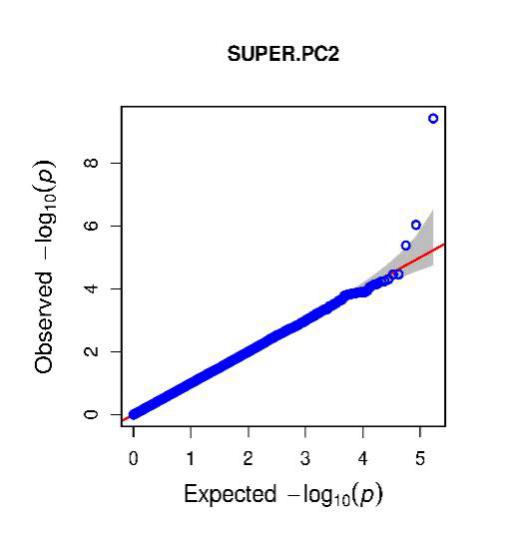 |  |  |

| **(a)** | **(b)** | **(c)** |
| --- | --- | --- |
| 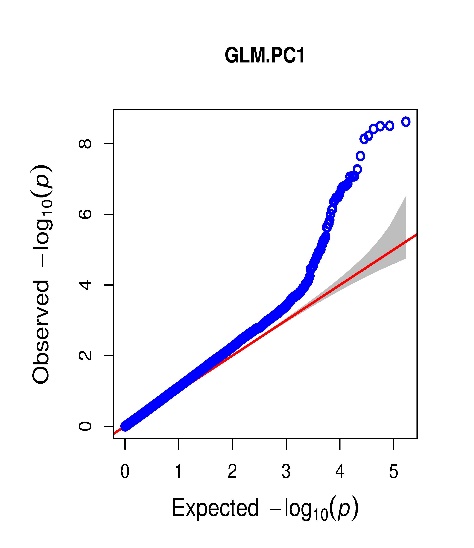 | 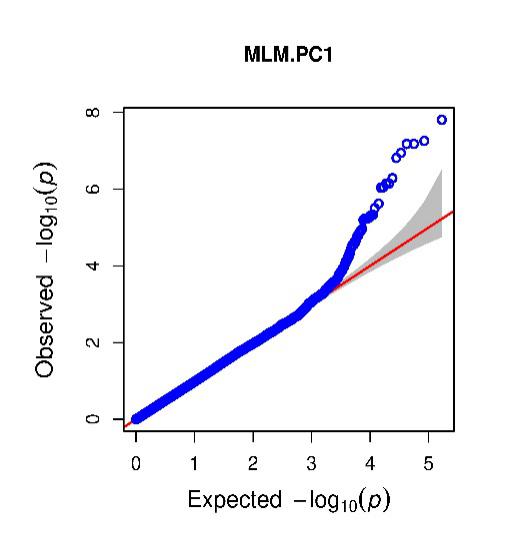 | 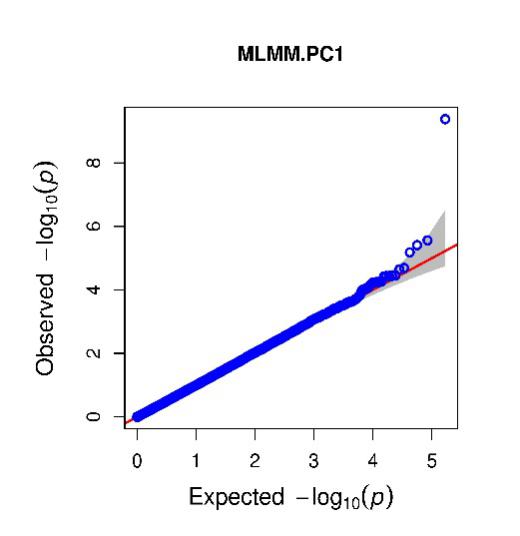 |
| **(d)** | **(e)** | **(f)** |
| 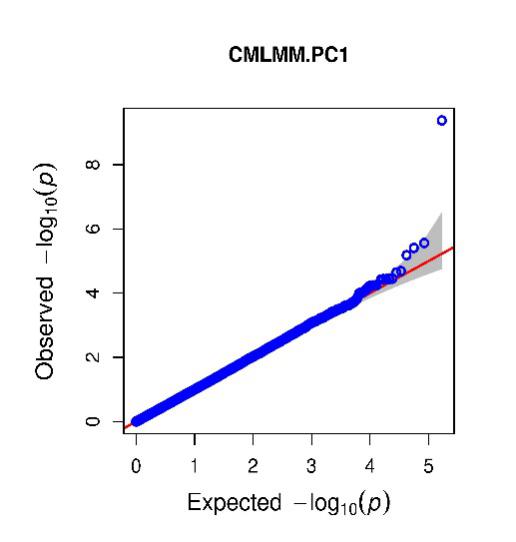 | 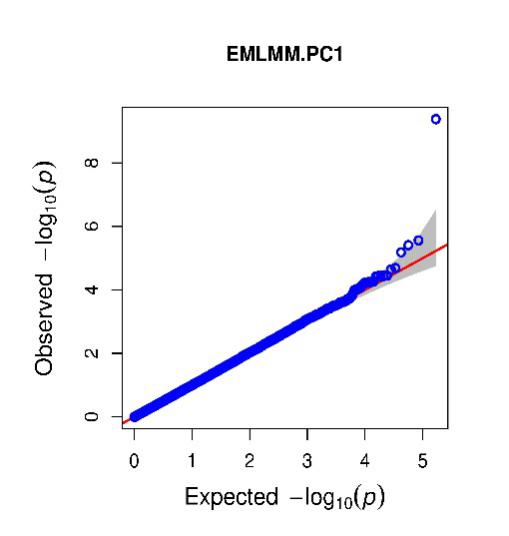 | 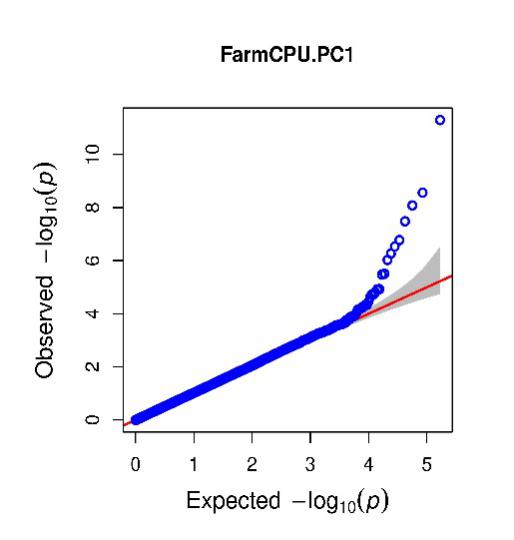 |
| **(g)** | **Figure S9:** Quantile-Quantile (Q-Q) plots based on GWAS results from different association models for PC1 in the year 2018-19. Model representations are GLM (a), MLM (b), MLMM (c), CMLM(d), ECMLM(e), FarmCPU(f) and SUPER(g). *x* axis plots expected –log_10_(*p*) values and *y* axis plots observed –log_10_(*p*) values respectively. | |
| 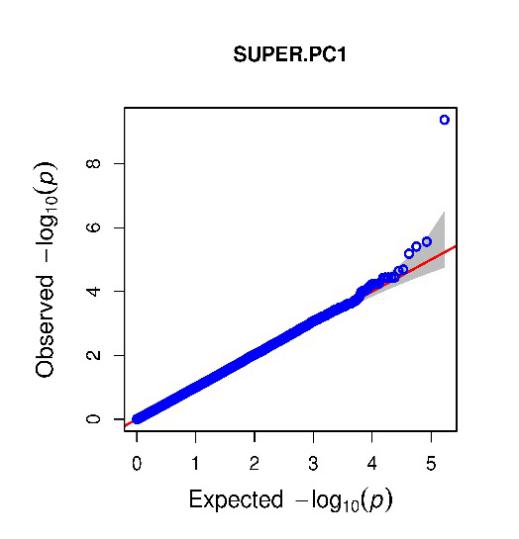 |  |  |

| **(a)** | **(b)** | **(c)** |
| --- | --- | --- |
| 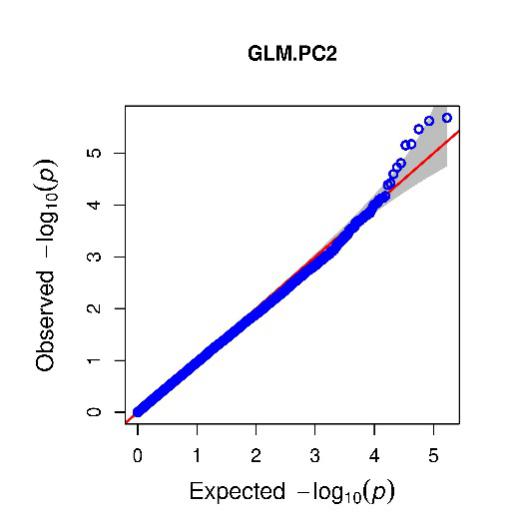 | 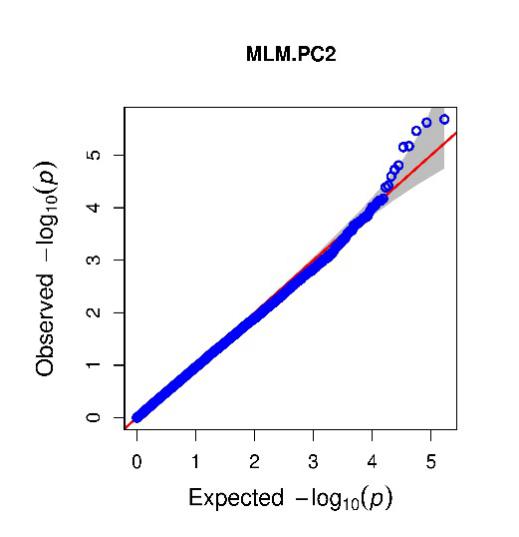 | 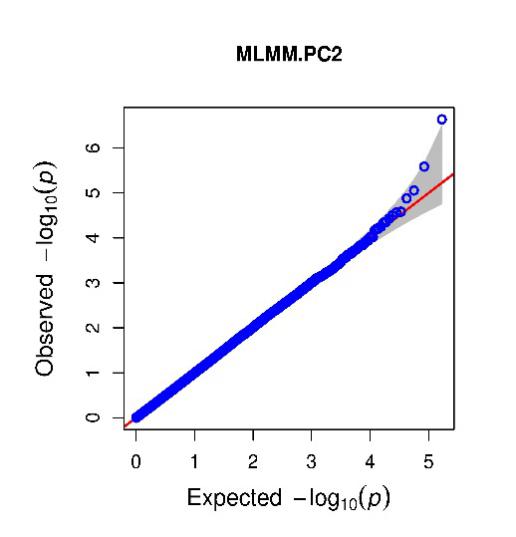 |
| **(d)** | **(e)** | **(f)** |
| 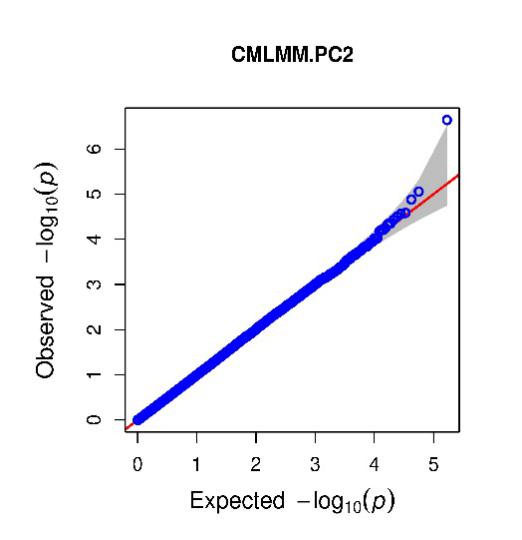 | 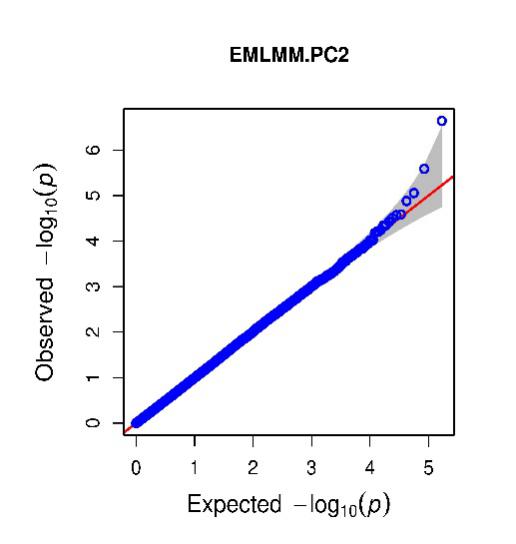 | 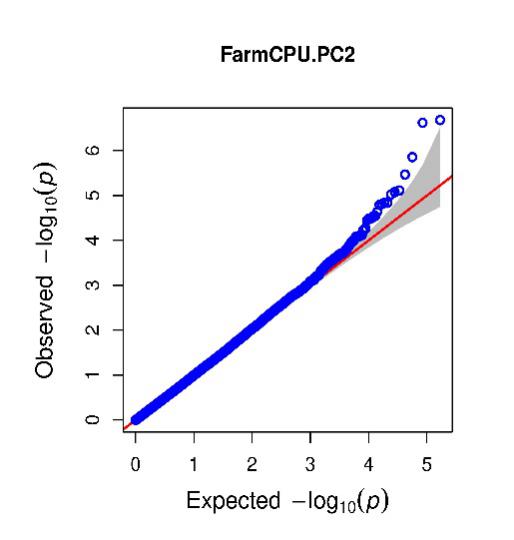 |
| **(g)** | **Figure S10:** Quantile-Quantile (Q-Q) plots based on GWAS results from different association models for PC2 in the year 2018-19. Model representations are GLM (a), MLM (b), MLMM (c), CMLM(d), ECMLM(e), FarmCPU(f) and SUPER(g). *x* axis plots expected –log_10_(*p*) values and *y* axis plots observed –log_10_(*p*) values respectively. | |
| 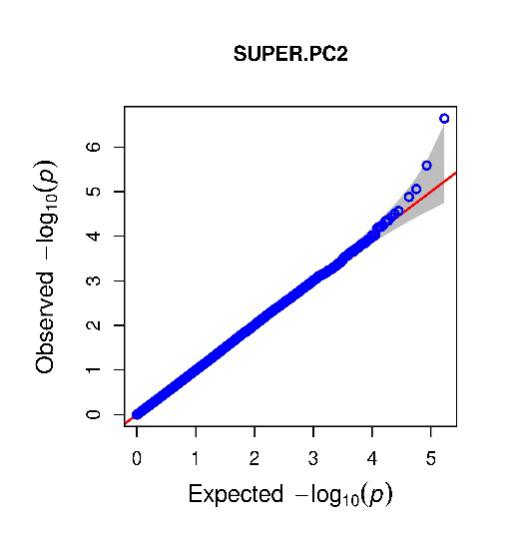 |  |  |

| 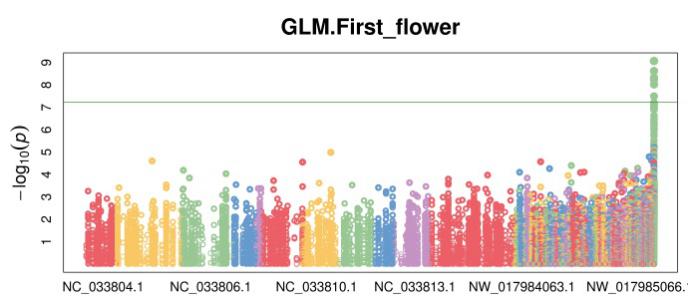 | 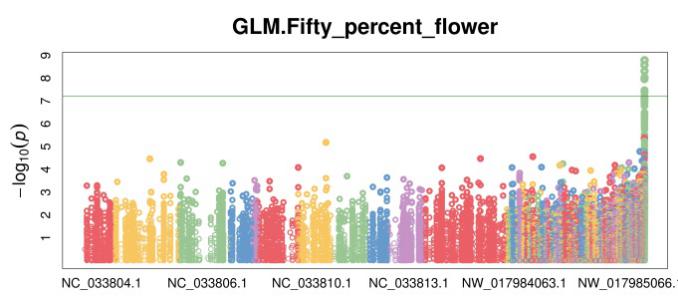 |
| --- | --- |
| 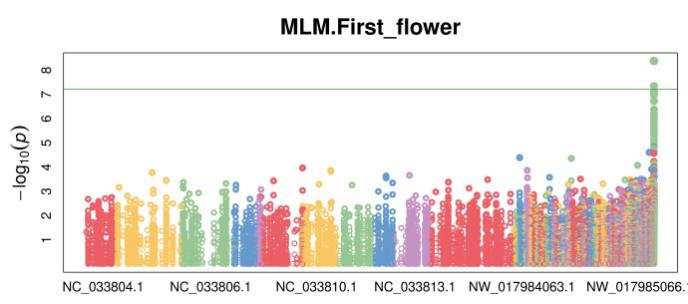 | 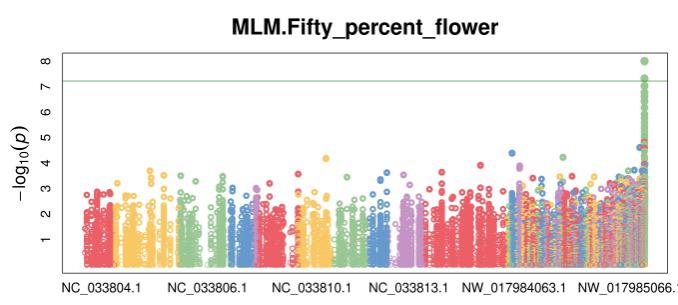 |
| 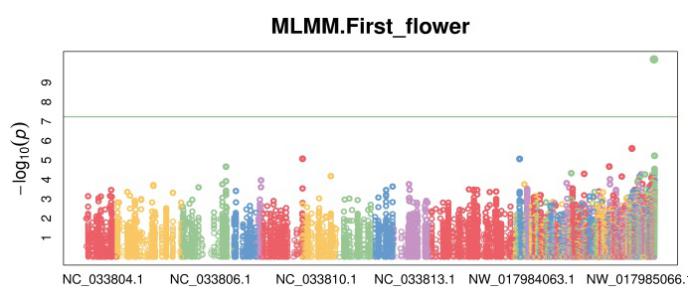 | 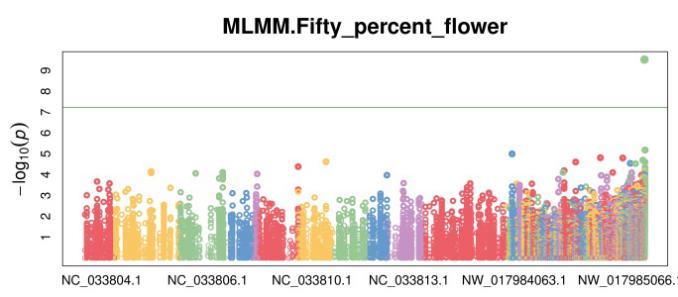 |
| 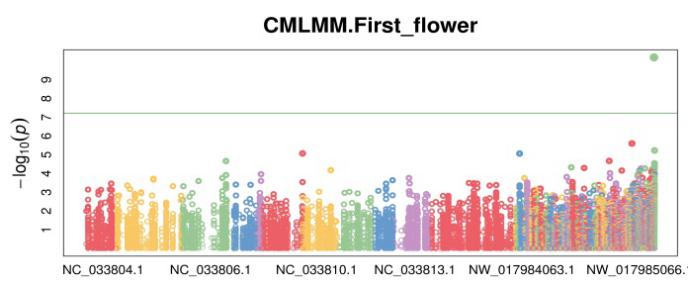 | 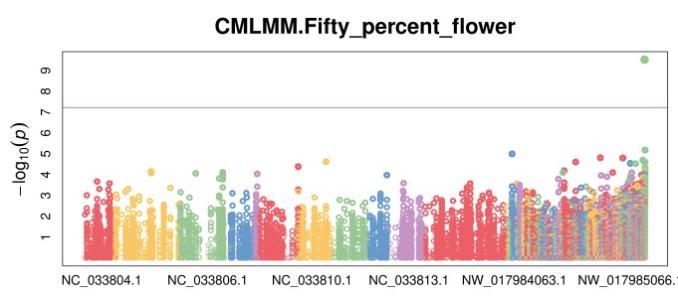 |
| 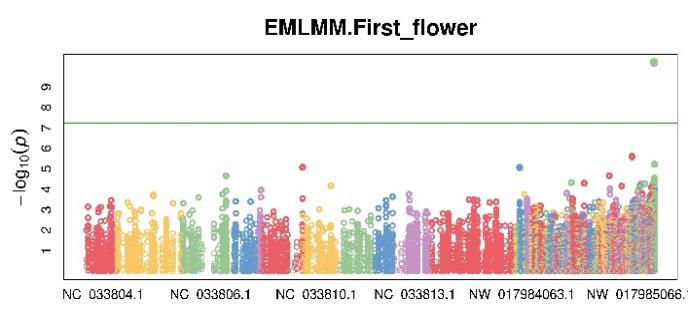 | 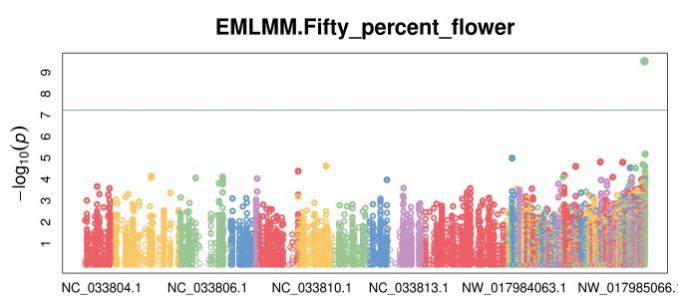 |
| 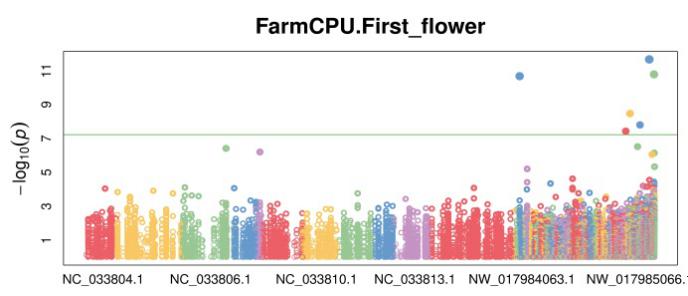 | 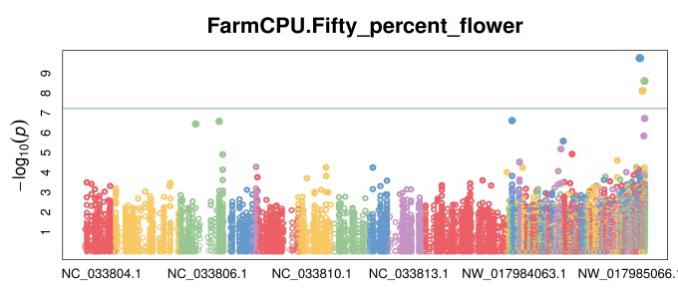 |
| 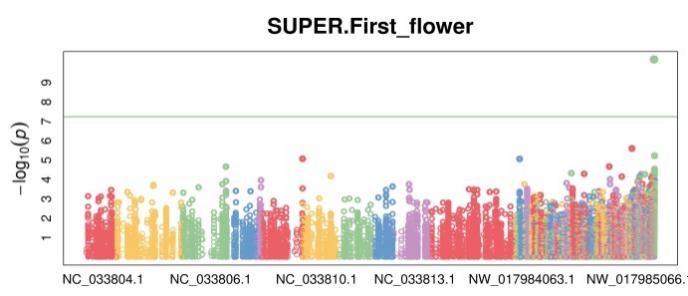 | 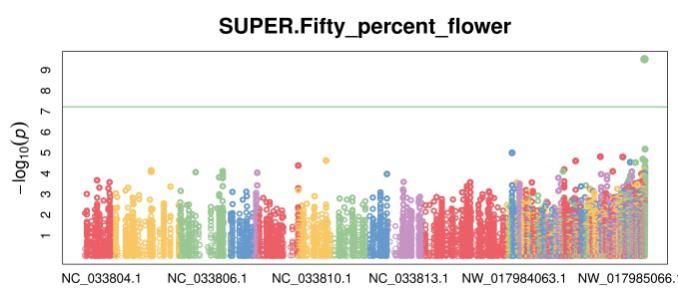 |

**Figure S11:** Manhattan plots for DOF (Left side) and DFF (Right side) for the year 2018-19. Top to bottom order is GLM, MLM, MLMM, CMLM, ECMLM, FarmCPU and SUPER.

| 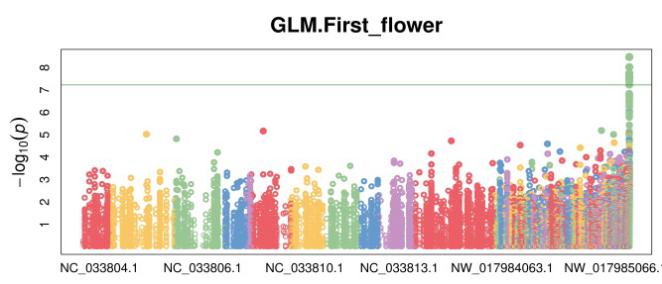 | 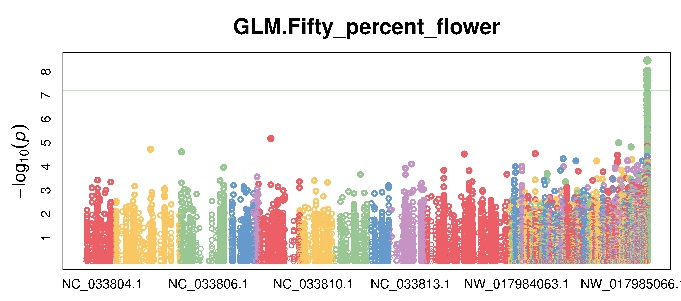 |
| --- | --- |
| 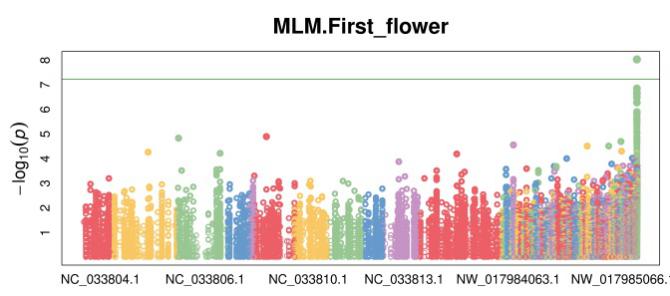 | 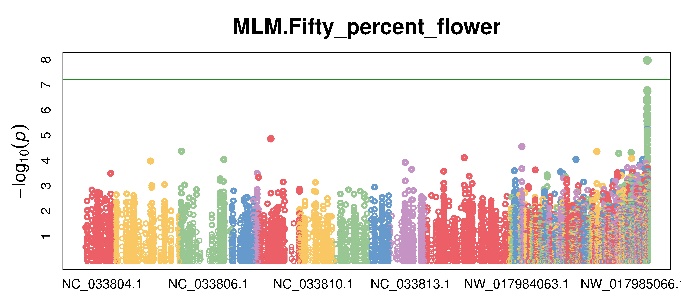 |
| 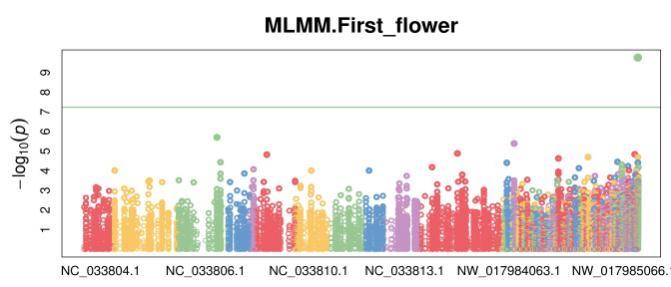 | 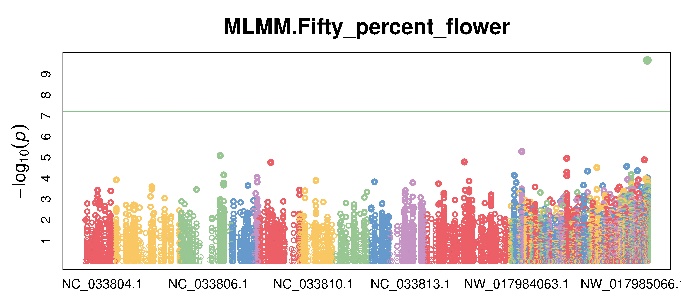 |
| 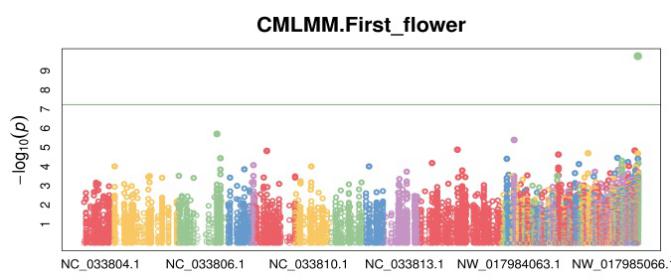 | 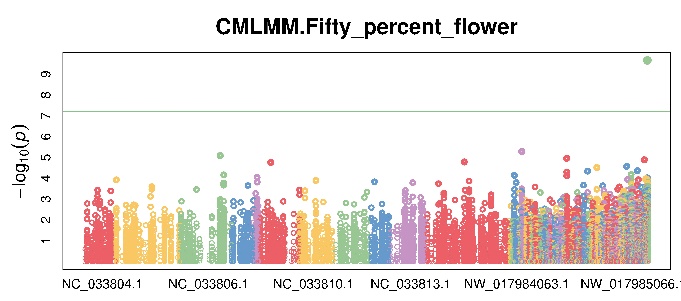 |
| 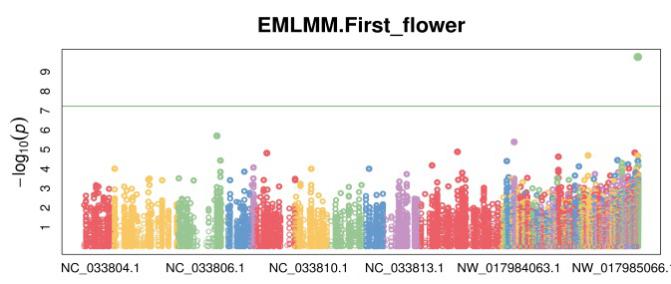 | 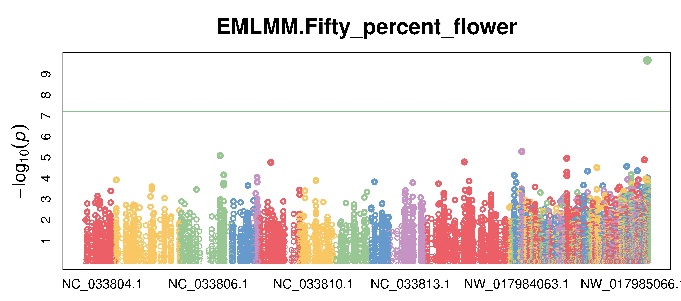 |
| 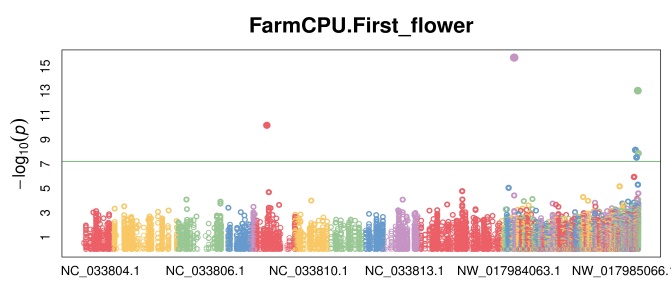 | 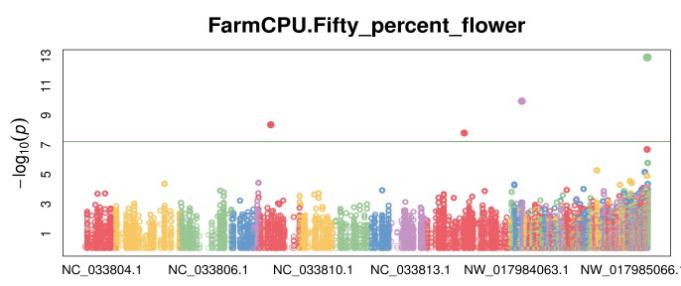 |
| 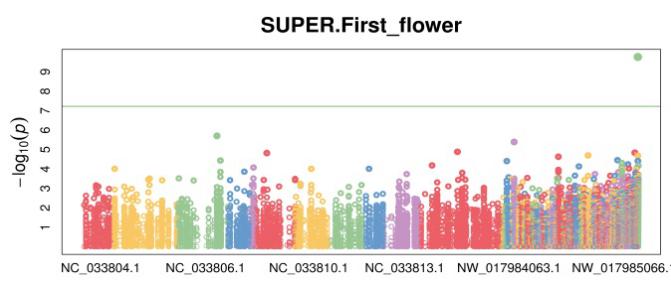 | 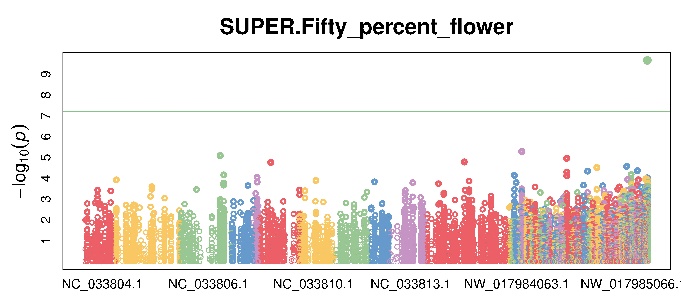 |

**Figure S12:** Manhattan plots for DOF (Left side) and DFF (Right side) for the year 2019-20. Top to bottom order is GLM, MLM, MLMM, CMLM, ECMLM, FarmCPU and SUPER.

| 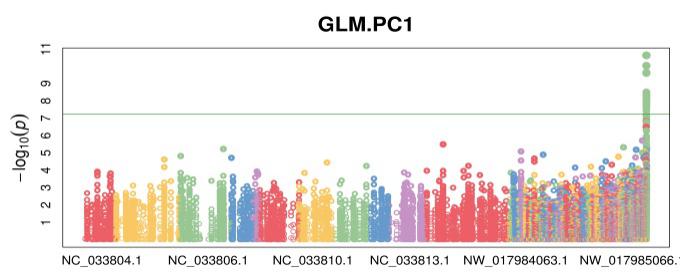 | 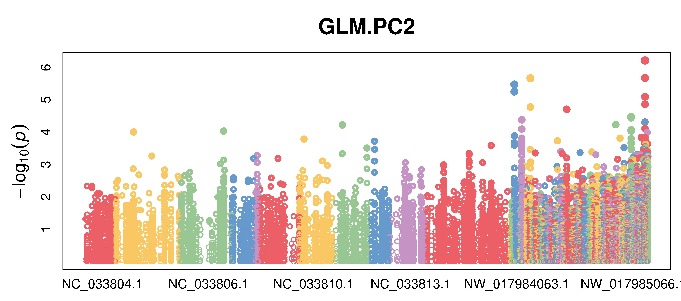 |
| --- | --- |
| 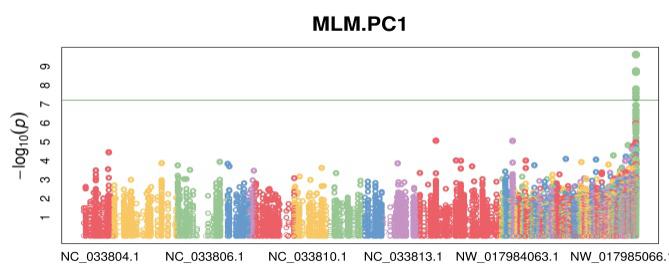 | 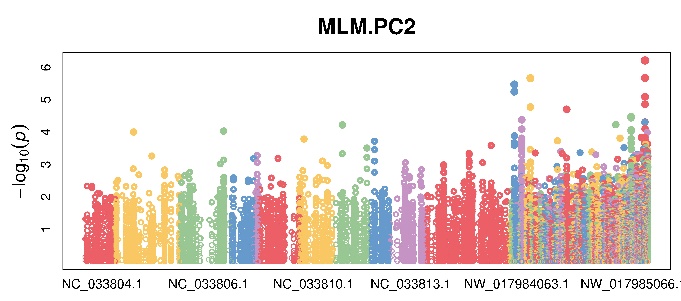 |
| 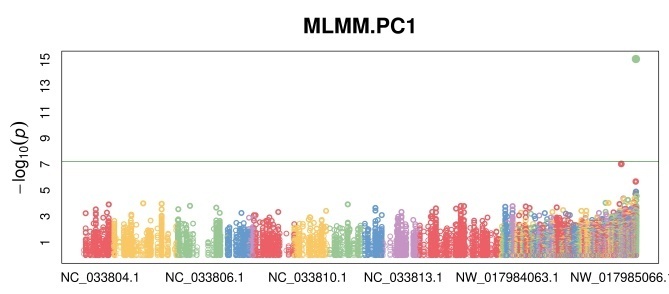 | 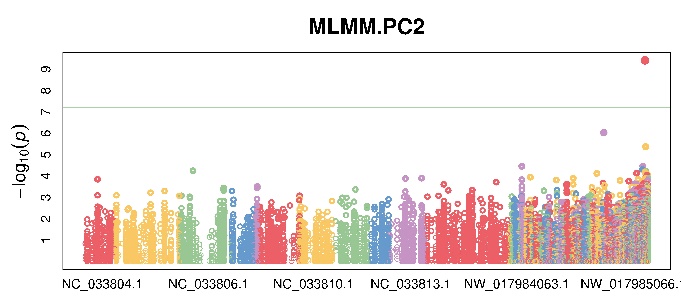 |
| 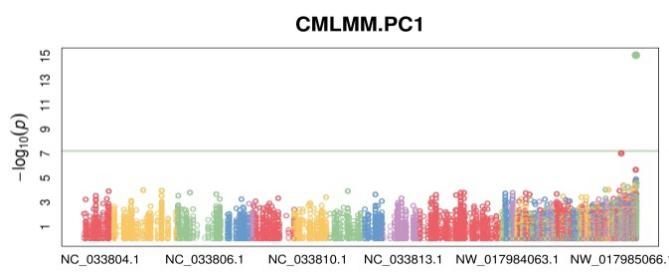 | 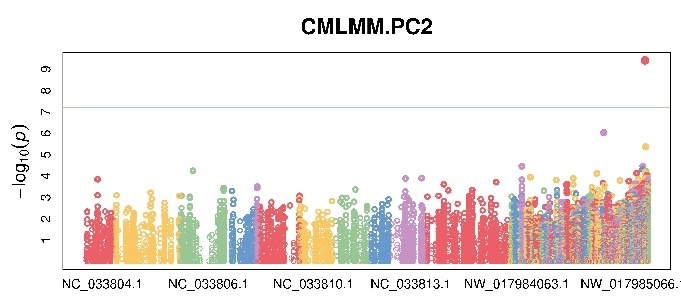 |
|  |  |
|  |  |
|  |  |

**Figure S13:** Manhattan plots for PC1 (Left side) and PC2 (Right side) for the year 2017-18. Top to bottom order is GLM, MLM, MLMM, CMLM, ECMLM, FarmCPU and SUPER.

|  |  |
| --- | --- |
|  |  |
|  |  |
|  |  |
|  |  |
|  |  |
|  |  |

**Figure S14:** Manhattan plots for PC1 (Left side) and PC2 (Right side) for the year 2018-19. Top to bottom order is GLM, MLM, MLMM, CMLM, ECMLM, FarmCPU and SUPER.

**Table S5:** Marker trait association as found for DOF (2017-18)

| Model | SNP | Chromosome | Position | FDR adjusted P value |
| --- | --- | --- | --- | --- |
| CMLM | 812679326:250:+ | NW_017988637.1 | 863 | 2.67564E-06 |
| EMLM | 812679326:250:+ | NW_017988637.1 | 863 | 2.67564E-06 |
| FarmCPU | 812678863:261:+ | NW_017988637.1 | 1117 | 9.76011E-13 |
| FarmCPU | 392468479:318:+ | NW_017984071.1 | 155917 | 3.53364E-05 |
| FarmCPU | 725832748:272:+ | NW_017985276.1 | 22384 | 3.53364E-05 |
| FarmCPU | 791831919:74:+ | NW_017986933.1 | 11488 | 0.000112893 |
| FarmCPU | 834373094:36:+ | NC_031429.1 | 45903 | 0.000150223 |
| FarmCPU | 142343707:25:+ | NC_033807.1 | 9366686 | 0.000747112 |
| GLM | 812678863:261:+ | NW_017988637.1 | 1117 | 1.43876E-05 |
| GLM | 812679326:250:- | NW_017988637.1 | 863 | 1.43876E-05 |
| GLM | 812678708:226:+ | NW_017988637.1 | 1004 | 2.28451E-05 |
| GLM | 812678807:41:+ | NW_017988637.1 | 869 | 0.000129716 |
| GLM | 812678873:263:+ | NW_017988637.1 | 1124 | 0.000129716 |
| GLM | 812678923:291:+ | NW_017988637.1 | 1177 | 0.000162732 |
| GLM | 812678650:310:+ | NW_017988637.1 | 1032 | 0.000169626 |
| GLM | 812679114:281:- | NW_017988637.1 | 703 | 0.000169715 |
| GLM | 812679132:4:- | NW_017988637.1 | 989 | 0.000218988 |
| GLM | 812679254:219:- | NW_017988637.1 | 835 | 0.000314499 |
| GLM | 812678903:74:+ | NW_017988637.1 | 950 | 0.00037627 |
| GLM | 812679356:256:- | NW_017988637.1 | 887 | 0.000441873 |
| GLM | 812678831:291:+ | NW_017988637.1 | 1131 | 0.000534902 |
| GLM | 812678781:295:+ | NW_017988637.1 | 1110 | 0.000637824 |
| MLM | 812679326:250:- | NW_017988637.1 | 863 | 0.000131143 |
| MLM | 812678863:261:+ | NW_017988637.1 | 1117 | 0.000131143 |
| MLM | 812678807:41:+ | NW_017988637.1 | 869 | 0.000667671 |
| MLM | 812678708:226:+ | NW_017988637.1 | 1004 | 0.001113215 |
| MLM | 812678650:310:+ | NW_017988637.1 | 1032 | 0.001350277 |
| MLM | 812679114:281:- | NW_017988637.1 | 703 | 0.001350277 |
| MLMM | 812679326:250:- | NW_017988637.1 | 863 | 2.67564E-06 |
| SUPER | 812679326:250:- | NW_017988637.1 | 863 | 2.67564E-06 |

**Table S6:** Marker trait association as found for DFF (2017-18)

| Model | SNP | Chromosome | Position | P value | FDR adjusted P values |
| --- | --- | --- | --- | --- | --- |
| CMLM | 812679326:250:- | NW_017988637.1 | 863 | 1.56358E-16 | 2.6353E-11 |
| EMLM | 812679326:250:- | NW_017988637.1 | 863 | 1.56358E-16 | 2.6353E-11 |
| FarmCPU | 812679326:250:- | NW_017988637.1 | 863 | 7.6136E-19 | 1.28E-13 |
| FarmCPU | 35373484:284:+ | NC_033805.1 | 6362335 | 1.55943E-10 | 1.31E-05 |
| GLM | 812679326:250:- | NW_017988637.1 | 863 | 2.43162E-11 | 4.0982E-06 |
| GLM | 812678863:261:+ | NW_017988637.1 | 1117 | 1.46681E-10 | 1.2361E-05 |
| GLM | 812678708:226:+ | NW_017988637.1 | 1004 | 2.94439E-10 | 1.6542E-05 |
| GLM | 812678873:263:+ | NW_017988637.1 | 1124 | 5.68579E-10 | 2.3957E-05 |
| GLM | 812679114:281:- | NW_017988637.1 | 703 | 1.09133E-09 | 3.6787E-05 |
| GLM | 812678781:295:+ | NW_017988637.1 | 1110 | 2.87224E-09 | 8.0681E-05 |
| GLM | 812678807:41:+ | NW_017988637.1 | 869 | 5.12511E-09 | 0.000122 |
| GLM | 806925787:88:+ | NW_017987977.1 | 2454 | 5.79078E-09 | 0.000122 |
| GLM | 812678903:74:+ | NW_017988637.1 | 950 | 6.57929E-09 | 0.00012321 |
| GLM | 812678650:310:+ | NW_017988637.1 | 1032 | 7.5321E-09 | 0.00012695 |
| GLM | 812679132:4:- | NW_017988637.1 | 989 | 9.45405E-09 | 0.00014485 |
| GLM | 812678923:291:+ | NW_017988637.1 | 1177 | 1.16408E-08 | 0.0001635 |
| GLM | 812678628:280:+ | NW_017988637.1 | 980 | 1.26497E-08 | 0.000164 |
| GLM | 812679356:256:- | NW_017988637.1 | 887 | 1.68751E-08 | 0.00020315 |
| GLM | 812678831:291:+ | NW_017988637.1 | 1131 | 2.54523E-08 | 0.00028598 |
| GLM | 812679254:219:- | NW_017988637.1 | 835 | 3.18802E-08 | 0.00033582 |
| GLM | 812678969:75:+ | NW_017988637.1 | 984 | 3.65658E-08 | 0.00036252 |
| GLM | 812679290:87:- | NW_017988637.1 | 990 | 4.09308E-08 | 0.00038325 |
| GLM | 812678959:7:+ | NW_017988637.1 | 911 | 5.18839E-08 | 0.00046024 |
| GLM | 812678857:270:+ | NW_017988637.1 | 1123 | 5.543E-08 | 0.0004631 |
| GLM | 812679407:82:- | NW_017988637.1 | 1112 | 5.77016E-08 | 0.0004631 |
| MLM | 812679326:250:- | NW_017988637.1 | 863 | 4.17603E-11 | 7.0383E-06 |
| MLM | 812678863:261:+ | NW_017988637.1 | 1117 | 1.4421E-09 | 0.00012153 |
| MLM | 812679114:281:- | NW_017988637.1 | 703 | 3.3781E-09 | 0.00018978 |
| MLM | 812678781:295:+ | NW_017988637.1 | 1110 | 5.50397E-09 | 0.00022987 |
| MLM | 812678708:226:+ | NW_017988637.1 | 1004 | 6.81958E-09 | 0.00022987 |
| MLM | 812678873:263:+ | NW_017988637.1 | 1124 | 1.64621E-08 | 0.00040603 |
| MLM | 812678807:41:+ | NW_017988637.1 | 869 | 1.68638E-08 | 0.00040603 |
| MLM | 812678650:310:+ | NW_017988637.1 | 1032 | 2.43894E-08 | 0.00051382 |
| MLMM | 812679326:250:- | NW_017988637.1 | 863 | 1.56358E-16 | 2.6353E-11 |
| SUPER | 812679326:250:- | NW_017988637.1 | 863 | 1.56358E-16 | 2.6353E-11 |

**Table S7:** Marker trait association as found for DOF (2018-19)

| Model | SNP | Chromosome | Position | P value | FDR adjusted P values |
| --- | --- | --- | --- | --- | --- |
| CMLM | 812678807:41:+ | NW_017988637.1 | 869 | 6.36688E-11 | 1.07307E-05 |
| EMLM | 812678807:41:+ | NW_017988637.1 | 869 | 6.37E-11 | 1.07307E-05 |
| FarmCPU | 760222832:55:+ | NW_017985856.1 | 27685 | 2.17007E-12 | 3.65744E-07 |
| FarmCPU | 812678807:41:+ | NW_017988637.1 | 869 | 1.65488E-11 | 1.19335E-06 |
| FarmCPU | 376936577:87:- | NW_017984062.1 | 168305 | 2.12416E-11 | 1.19335E-06 |
| FarmCPU | 652249420:11:+ | NW_017984675.1 | 23436 | 3.39832E-09 | 0.000143188 |
| FarmCPU | 709017214:7:- | NW_017985090.1 | 533 | 1.58876E-08 | 0.000535539 |
| FarmCPU | 633271872:58:+ | NW_017984581.1 | 74012 | 3.7203E-08 | 0.001045033 |
| GLM | 812678807:41:+ | NW_017988637.1 | 869 | 8.46635E-10 | 0.000142692 |
| GLM | 812678650:310:+ | NW_017988637.1 | 1032 | 2.37792E-09 | 0.000200388 |
| GLM | 812678863:261:+ | NW_017988637.1 | 1117 | 4.68988E-09 | 0.000242692 |
| GLM | 812678708:226:+ | NW_017988637.1 | 1004 | 5.75987E-09 | 0.000242692 |
| GLM | 812679326:250:- | NW_017988637.1 | 863 | 9.46748E-09 | 0.00031913 |
| GLM | 812678781:295:+ | NW_017988637.1 | 1110 | 3.17729E-08 | 0.000892502 |
| MLM | 812678807:41:+ | NW_017988637.1 | 869 | 4.14385E-09 | 0.000698404 |
| MLM | 812678650:310:+ | NW_017988637.1 | 1032 | 4.49276E-08 | 0.003786047 |
| MLMM | 812678807:41:+ | NW_017988637.1 | 869 | 6.36688E-11 | 1.07307E-05 |
| SUPER | 812678807:41:+ | NW_017988637.1 | 869 | 6.36688E-11 | 1.07307E-05 |

**Table S8:** Marker trait association as found for DFF (2018-19)

| Model | SNP | Chromosome | Position | P value | FDR adjusted P values |
| --- | --- | --- | --- | --- | --- |
| CMLMM | 812678807:41:+ | NW_017988637.1 | 869 | 2.97657E-10 | 5.02E-05 |
| EMLMM | 812678807:41:+ | NW_017988637.1 | 869 | 2.98E-10 | 5.02E-05 |
| FarmCPU | 760222832:55:+ | NW_017985856.1 | 27685 | 1.72E-10 | 2.90E-05 |
| FarmCPU | 812678807:41:+ | NW_017988637.1 | 869 | 2.43E-09 | 2.05E-04 |
| FarmCPU | 785047004:88:+ | NW_017986607.1 | 3977 | 7.57E-09 | 4.25E-04 |
| GLM | 812678807:41:+ | NW_017988637.1 | 869 | 1.61E-09 | 0.00022181 |
| GLM | 812678650:310:+ | NW_017988637.1 | 1032 | 2.63E-09 | 0.00022181 |
| GLM | 812678863:261:+ | NW_017988637.1 | 1117 | 4.87E-09 | 0.00027344 |
| GLM | 812679326:250:- | NW_017988637.1 | 863 | 9.32E-09 | 0.00036948 |
| GLM | 812678708:226:+ | NW_017988637.1 | 1004 | 1.10E-08 | 0.00036948 |
| GLM | 812678857:270:+ | NW_017988637.1 | 1123 | 3.37E-08 | 0.00094682 |
| GLM | 812679114:281:- | NW_017988637.1 | 703 | 4.13E-08 | 0.00099447 |
| MLM | 812678807:41:+ | NW_017988637.1 | 869 | 9.88E-09 | 0.001666 |
| MLM | 812678650:310:+ | NW_017988637.1 | 1032 | 4.70E-08 | 0.00396103 |
| MLMM | 812678807:41:+ | NW_017988637.1 | 869 | 2.98E-10 | 5.0167E-05 |
| SUPER | 812678807:41:+ | NW_017988637.1 | 869 | 2.98E-10 | 5.0167E-05 |

**Table S9:** Marker trait association as found for DOF (2019-20)

| Model | SNP | Chromosome | Position | P value | FDR adjusted P values |
| --- | --- | --- | --- | --- | --- |
| CMLM | 812678807:41:+ | NW_017988637.1 | 869 | 1.72E-10 | 2.89888E-05 |
| EMLM | 812678807:41:+ | NW_017988637.1 | 869 | 1.72E-10 | 2.89888E-05 |
| FarmCPU | 392479221:11:+ | NW_017984071.1 | 161167 | 1.86E-16 | 3.13604E-11 |
| FarmCPU | 812678807:41:+ | NW_017988637.1 | 869 | 9.57E-14 | 8.0641E-09 |
| FarmCPU | 164755426:80:+ | NC_033809.1 | 6932346 | 6.62E-11 | 3.71774E-06 |
| FarmCPU | 781124881:96:- | NW_017986454.1 | 6679 | 7.06E-09 | 0.000297314 |
| FarmCPU | 834384838:29:- | NC_031429.1 | 64092 | 1.31E-08 | 0.000442659 |
| FarmCPU | 791831919:74:+ | NW_017986933.1 | 11488 | 2.87E-08 | 0.000806471 |
| GLM | 812678807:41:+ | NW_017988637.1 | 869 | 3.33E-09 | 0.00056188 |
| GLM | 812679132:61:- | NW_017988637.1 | 932 | 9.41E-09 | 0.000793056 |
| GLM | 812678650:310:+ | NW_017988637.1 | 1032 | 1.78E-08 | 0.000859388 |
| GLM | 812678719:319:+ | NW_017988637.1 | 1103 | 2.46E-08 | 0.000859388 |
| GLM | 812679132:4:- | NW_017988637.1 | 989 | 2.78E-08 | 0.000859388 |
| GLM | 812678919:31:+ | NW_017988637.1 | 915 | 3.06E-08 | 0.000859388 |
| GLM | 812678863:261:+ | NW_017988637.1 | 1117 | 4.00E-08 | 0.000963408 |
| GLM | 812678903:74:+ | NW_017988637.1 | 950 | 5.82E-08 | 0.001225922 |
| MLM | 812678807:41:+ | NW_017988637.1 | 869 | 9.25E-09 | 0.001558465 |
| MLMM | 812678807:41:+ | NW_017988637.1 | 869 | 1.72E-10 | 2.89888E-05 |
| SUPER | 812678807:41:+ | NW_017988637.1 | 869 | 1.72E-10 | 2.89888E-05 |

**Table S10:** Marker trait association as found for DFF (2019-20)

| Model | SNP | Chromosome | Position | P value | FDR adjusted P values |
| --- | --- | --- | --- | --- | --- |
| CMLM | 812678807:41:+ | NW_017988637.1 | 869 | 2.1612E-10 | 3.6425E-05 |
| EMLM | 812678807:41:+ | NW_017988637.1 | 869 | 2.1612E-10 | 3.6425E-05 |
| FarmCPU | 812678807:41:+ | NW_017988637.1 | 869 | 1.22285E-13 | 2.061E-08 |
| FarmCPU | 392479221:11:+ | NW_017984071.1 | 161167 | 1.10428E-10 | 9.3058E-06 |
| FarmCPU | 164755426:80:+ | NC_033809.1 | 6932346 | 4.33088E-09 | 0.00024331 |
| FarmCPU | 330539130:289:+ | NC_033814.1 | 21328862 | 1.57267E-08 | 0.00066265 |
| GLM | 812678807:41:+ | NW_017988637.1 | 869 | 3.30822E-09 | 0.00055757 |
| GLM | 812679132:61:- | NW_017988637.1 | 932 | 9.14771E-09 | 0.00073346 |
| GLM | 812678650:310:+ | NW_017988637.1 | 1032 | 1.30556E-08 | 0.00073346 |
| GLM | 812678919:31:+ | NW_017988637.1 | 915 | 2.01068E-08 | 0.00081456 |
| GLM | 812679132:4:- | NW_017988637.1 | 989 | 2.41652E-08 | 0.00081456 |
| GLM | 812678719:319:+ | NW_017988637.1 | 1103 | 3.78123E-08 | 0.00103289 |
| GLM | 812678863:261:+ | NW_017988637.1 | 1117 | 4.2899E-08 | 0.00103289 |
| MLM | 812678807:41:+ | NW_017988637.1 | 869 | 1.056E-08 | 0.00177979 |
| MLMM | 812678807:41:+ | NW_017988637.1 | 869 | 2.1612E-10 | 3.6425E-05 |
| SUPER | 812678807:41:+ | NW_017988637.1 | 869 | 2.16E-10 | 3.6425E-05 |

**Table S11:** Marker trait association as found for PC1 (2017-18)

| Model | SNP | Chromosome | Position | P value | FDR adjusted P values |
| --- | --- | --- | --- | --- | --- |
| CMLM | 812679326:250:- | NW_017988637.1 | 863 | 8.12499E-16 | 1.36939E-10 |
| EMLM | 812679326:250:- | NW_017988637.1 | 863 | 8.12499E-16 | 1.36939E-10 |
| FarmCPU | 21256769:324:+ | NC_033804.1 | 14401967 | 7.61886E-09 | 0.000787591 |
| FarmCPU | 812679326:250:- | NW_017988637.1 | 863 | 9.34604E-09 | 0.000787591 |
| FarmCPU | 740074801:308:- | NW_017985477.1 | 249 | 2.57697E-08 | 0.001447741 |
| GLM | 812679326:250:- | NW_017988637.1 | 863 | 2.40563E-11 | 4.05445E-06 |
| GLM | 812678863:261:+ | NW_017988637.1 | 1117 | 9.42087E-11 | 7.93896E-06 |
| GLM | 812678708:226:+ | NW_017988637.1 | 1004 | 2.34483E-10 | 1.05445E-05 |
| GLM | 812679114:281:- | NW_017988637.1 | 703 | 2.50255E-10 | 1.05445E-05 |
| GLM | 812678781:295:+ | NW_017988637.1 | 1110 | 3.15979E-09 | 0.00010651 |
| GLM | 812678650:310:+ | NW_017988637.1 | 1032 | 4.56058E-09 | 0.000128107 |
| GLM | 812678873:263:+ | NW_017988637.1 | 1124 | 5.81841E-09 | 0.000140091 |
| GLM | 812678807:41:+ | NW_017988637.1 | 869 | 7.32391E-09 | 0.000151189 |
| GLM | 812679132:4:- | NW_017988637.1 | 989 | 8.07344E-09 | 0.000151189 |
| GLM | 812678923:291:+ | NW_017988637.1 | 1177 | 1.11804E-08 | 0.000182673 |
| GLM | 812678628:280:+ | NW_017988637.1 | 980 | 1.19224E-08 | 0.000182673 |
| GLM | 812678903:74:+ | NW_017988637.1 | 950 | 1.65587E-08 | 0.000232566 |
| GLM | 812679356:256:- | NW_017988637.1 | 887 | 1.88775E-08 | 0.000244739 |
| GLM | 812678831:291:+ | NW_017988637.1 | 1131 | 2.48916E-08 | 0.00028352 |
| GLM | 812679132:61:- | NW_017988637.1 | 932 | 2.52332E-08 | 0.00028352 |
| GLM | 812679254:219:- | NW_017988637.1 | 835 | 3.59556E-08 | 0.000378747 |
| MLM | 812679326:250:- | NW_017988637.1 | 863 | 2.32821E-10 | 3.92396E-05 |
| MLM | 812679114:281:- | NW_017988637.1 | 703 | 1.70588E-09 | 0.000113884 |
| MLM | 812678863:261:+ | NW_017988637.1 | 1117 | 2.02712E-09 | 0.000113884 |
| MLM | 812678708:226:+ | NW_017988637.1 | 1004 | 1.53526E-08 | 0.000646882 |
| MLM | 812678781:295:+ | NW_017988637.1 | 1110 | 2.43339E-08 | 0.000820249 |
| MLM | 812678807:41:+ | NW_017988637.1 | 869 | 3.87416E-08 | 0.001088252 |
| MLM | 812678650:310:+ | NW_017988637.1 | 1032 | 4.65694E-08 | 0.001121258 |
| MLMM | 812679326:250:- | NW_017988637.1 | 863 | 8.12499E-16 | 1.36939E-10 |
| SUPER | 812679326:250:- | NW_017988637.1 | 863 | 8.12499E-16 | 1.36939E-10 |

**Table S12:** Marker trait association as found for PC1 (2018-19)

| Model | SNP | Chromosome | Position | P value | FDR adjusted P values |
| --- | --- | --- | --- | --- | --- |
| CMLM | 812678807:41:+ | NW_017988637.1 | 869 | 4.17129E-10 | 7.0303E-05 |
| EMLM | 812678807:41:+ | NW_017988637.1 | 869 | 4.17129E-10 | 7.0303E-05 |
| FarmCPU | 392479221:11:+ | NW_017984071.1 | 161167 | 5.05809E-12 | 8.5249E-07 |
| FarmCPU | 324910270:94:- | NC_033814.1 | 17612083 | 2.74792E-09 | 0.000231567 |
| FarmCPU | 760222832:55:+ | NW_017985856.1 | 27685 | 8.33453E-09 | 0.000468234 |
| FarmCPU | 593701379:271:+ | NW_017984430.1 | 87462 | 3.30588E-08 | 0.001392933 |
| GLM | 812679326:250:- | NW_017988637.1 | 863 | 2.39738E-09 | 0.000161605 |
| GLM | 812678807:41:+ | NW_017988637.1 | 869 | 3.11459E-09 | 0.000161605 |
| GLM | 812678650:310:+ | NW_017988637.1 | 1032 | 3.18688E-09 | 0.000161605 |
| GLM | 812678863:261:+ | NW_017988637.1 | 1117 | 3.83542E-09 | 0.000161605 |
| GLM | 812678708:226:+ | NW_017988637.1 | 1004 | 5.99504E-09 | 0.000202081 |
| GLM | 812679114:281:- | NW_017988637.1 | 703 | 7.32446E-09 | 0.000205744 |
| GLM | 812678781:295:+ | NW_017988637.1 | 1110 | 2.23525E-08 | 0.000538183 |
| GLM | 812678903:74:+ | NW_017988637.1 | 950 | 5.34319E-08 | 0.001125676 |
| MLM | 812678807:41:+ | NW_017988637.1 | 869 | 1.53934E-08 | 0.002594411 |
| MLM | 812679326:250:- | NW_017988637.1 | 863 | 5.47256E-08 | 0.002776289 |
| MLMM | 812678807:41:+ | NW_017988637.1 | 869 | 4.17129E-10 | 7.0303E-05 |
| SUPER | 812678807:41:+ | NW_017988637.1 | 869 | 4.17129E-10 | 7.0303E-05 |

**Table S13:** Marker trait association as found for PC2 (2017-18)

| Model | SNP | Chromosome | Position | P value | FDR adjusted P values |
| --- | --- | --- | --- | --- | --- |
| CMLMM | 781824196:43:+ | NW_017986481.1 | 4126 | 3.81554E-10 | 6.43072E-05 |
| EMLMM | 781824196:43:+ | NW_017986481.1 | 4126 | 3.81554E-10 | 6.43072E-05 |
| FARMcpu | 786499924:273:+ | NW_017986665.1 | 6570 | 3.78504E-12 | 3.9501E-07 |
| FARMcpu | 812976342:343:+ | NW_017988680.1 | 3573 | 4.68744E-12 | 3.9501E-07 |
| FARMcpu | 788430456:86:+ | NW_017986752.1 | 18630 | 3.21745E-10 | 1.80756E-05 |
| FARMcpu | 801404902:13:- | NW_017987505.1 | 2550 | 3.5706E-09 | 1.50447E-04 |
| FARMcpu | 677450242:4:- | NW_017984821.1 | 61153 | 5.09337E-08 | 1.71687E-03 |
| MLMM | 781824196:43:+ | NW_017986481.1 | 4126 | 3.81554E-10 | 6.43072E-05 |
| SUPER | 781824196:43:+ | NW_017986481.1 | 4126 | 3.81554E-10 | 6.43072E-05 |
